# Metabolism of Phosphatidylinositol 4-Kinase IIIα-Dependent PI4P Is Subverted by HCV and Is Targeted by a 4-Anilino Quinazoline with Antiviral Activity

**DOI:** 10.1371/journal.ppat.1002576

**Published:** 2012-03-08

**Authors:** Annalisa Bianco, Veronica Reghellin, Lorena Donnici, Simone Fenu, Reinaldo Alvarez, Chiara Baruffa, Francesco Peri, Massimiliano Pagani, Sergio Abrignani, Petra Neddermann, Raffaele De Francesco

**Affiliations:** 1 Department of Genomics and Molecular Biology, Virology Program, Istituto Nazionale Genetica Molecolare (INGM), Milano, Italy; 2 Department of Biotechnology and Biosciences, University of Milano Bicocca, Milano, Italy; University of Kentucky College of Medicine, United States of America

## Abstract

4-anilino quinazolines have been identified as inhibitors of HCV replication. The target of this class of compounds was proposed to be the viral protein NS5A, although unequivocal proof has never been presented. A 4-anilino quinazoline moiety is often found in kinase inhibitors, leading us to formulate the hypothesis that the anti-HCV activity displayed by these compounds might be due to inhibition of a cellular kinase. Type III phosphatidylinositol 4-kinase α (PI4KIIIα) has recently been identified as a host factor for HCV replication. We therefore evaluated AL-9, a compound prototypical of the 4-anilino quinazoline class, on selected phosphatidylinositol kinases. AL-9 inhibited purified PI4KIIIα and, to a lesser extent, PI4KIIIβ. In Huh7.5 cells, PI4KIIIα is responsible for the phosphatidylinositol-4 phosphate (PI4P) pool present in the plasma membrane. Accordingly, we observed a gradual decrease of PI4P in the plasma membrane upon incubation with AL-9, indicating that this agent inhibits PI4KIIIα also in living cells. Conversely, AL-9 did not affect the level of PI4P in the Golgi membrane, suggesting that the PI4KIIIβ isoform was not significantly inhibited under our experimental conditions. Incubation of cells expressing HCV proteins with AL-9 induced abnormally large clusters of NS5A, a phenomenon previously observed upon silencing PI4KIIIα by RNA interference. In light of our findings, we propose that the antiviral effect of 4-anilino quinazoline compounds is mediated by the inhibition of PI4KIIIα and the consequent depletion of PI4P required for the HCV membranous web. In addition, we noted that HCV has a profound effect on cellular PI4P distribution, causing significant enrichment of PI4P in the HCV-membranous web and a concomitant depletion of PI4P in the plasma membrane. This observation implies that HCV – by recruiting PI4KIIIα in the RNA replication complex – hijacks PI4P metabolism, ultimately resulting in a markedly altered subcellular distribution of the PI4KIIIα product.

## Author Summary

It is estimated that 3% of the world's population are chronically infected by the hepatitis C virus (HCV). Most infections become chronic and eventually evolve into cirrhosis and hepatocellular carcinoma. Host factors are interesting targets for anti-HCV therapies due to their inherent high genetic barrier to resistance. Recently, phosphatidylinositol 4-kinase α (PI4KIIIα) has been identified as a crucial host factor for HCV replication. Many different pathogens, including HCV, subvert components of the phosphatidylinositol-4 phosphate (PI4P) pathway to function in favor of their own life cycle. In this paper, we show that HCV dramatically alters cellular PI4P metabolism and distribution, resulting in the enrichment of PI4P in the membranous web required for viral replication with a concomitant decrease of PI4P in the plasma-membrane. Moreover, we demonstrate that 4-anilino quinazolines, antiviral agents previously believed to target HCV NS5A, do in fact inhibit PI4P formation by inhibition of PI4KIIIα. This compound class is a promising lead for the development of a novel antiviral therapy based on PI4KIIIα inhibition. Specific PI4KIIIα inhibitors would also be important research tools required for a deeper understanding of the functions and regulation of PI4P.

## Introduction

Hepatitis C virus (HCV) is an enveloped, single-stranded RNA virus classified as member of the Hepacivirus genus within the Flaviviridae family. The 9.6 kb positive-sense RNA genome contains a single open reading frame encoding a polyprotein of about 3,000 amino acids, flanked by highly structured 5′ and 3′ untranslated (UTR) regions. Following its release into the cytoplasm of the host cell, viral RNA is translated via an internal ribosome entry site (IRES), giving rise to a single polypeptide that is cleaved into 10 different mature protein products: Core, gpE1, gpE2, p7, NS2, NS3, NS4A, NS4B, NS5A, and NS5B. HCV RNA replication takes place in the cytoplasm, in association with a virus-induced intracellular membrane structure termed “membranous web”, onto which NS proteins assemble to form the so-called RNA replication complexes.

It is estimated that 3% of the world's population are chronically infected by the hepatitis C virus (HCV). Most infections become chronic and over time evolve into chronic hepatitis. The most unwanted complication of chronic hepatitis is cirrhosis, a massive liver fibrosis, which can lead to liver failure and hepatocellular carcinoma.

Since the discovery of hepatitis C virus (HCV) in the late 1980's much progress has been made in the understanding of the viral life cycle of HCV. Nonetheless, to date no vaccines are available and the current standard of care, involving lengthy treatment with a combination of ribavirin and pegylated interferon-α (peg-IFN-α), eradicates the infection in half of treated patients. A large effort has been made in the past two decades in order to develop novel anti-HCV therapies with greater efficacy. Two oral direct-acting antiviral agents (DAA) targeting the HCV NS3/4 protease, boceprevir and telaprevir, have recently reached the market and more are being developed [1]. While the initial efforts to the discovery of DAA focused almost exclusively on the best characterized HCV enzymes required for viral replication – the NS3/4A protease and the NS5B polymerase – in the past few years the NS5A viral protein has been attracting more and more attention as a target for drug development [1], [2]. NS5A possesses no known enzymatic activity. It is a multifunctional non-structural protein important for viral replication [3]–[6] as well as viral assembly [7]–[9]. It is a phosphoprotein consisting of three domains [10]. Domain I is highly conserved and forms a dimeric structure [11], [12], whereas domains II and III are believed to adopt a “natively unfolded” conformation [13], [14].

In recent years, several anti-HCV compounds identified using cell-based replicon screens were indicated to target NS5A based on the analysis of the mutations associated with emergence of resistance in the replicon system [15]–[17].

The most studied series of these “NS5A inhibitors” is represented by BMS-790052, an agent that is leading the field, having demonstrated potent antiviral activity in clinical studies [18]. Compounds in this class are characterized by a complex, dimeric or pseudo-dimeric structure and a high molecular weight, when compared with conventional “drug-like” small molecules [17], [19]. Resistance mutations against these compounds emerge readily in domain I of NS5A [20], with the most recurrent of these changes corresponding to variant of tyrosine at position 93 [20]. Although direct interaction with purified NS5A has not been demonstrated, compelling reverse genetic experiments [20] as well as molecular models [15], [21] strongly support the notion that NS5A is the direct target of these compounds.

A less characterized series of compounds, belonging to a different chemical class, was also initially indicated to target NS5A on the basis of the mutation pattern observed in resistant replicons [21]. The common structural element of this latter class of inhibitors is a 4-anilino quinazoline core. A representative member of this class of compounds is A-831/AZD-2836, an experimental antiviral agent that entered clinical trials but was later discontinued due to the lack of adequate exposure [17]. For these agents, the mutations reported to be associated with resistance were found to be different from those expected for the NS5A inhibitor described above, pointing to a different mechanism of action: a few mutations were found at the C-terminal end of NS5A domain I (E212D, L199F and T200P), whereas most mutations occurred in NS5A domains II and III (P299L, S370P, V388D, V362A, S390G and S370P). Additional mutations were also found in NS4B (S258T) and NS5B (S76A) [17], [21], [22]. Reverse genetics studies in which these mutations were reintroduced in the replicon, however, did not recapitulate the resistant phenotype observed in the original cellular clones [17], leaving thus the possibility open that these compounds act through a different viral or cellular target.

Interestingly, many kinase inhibitors, including some approved antitumoral drugs (gefitinib, lapatinib, erlotinib) are 4-anilino quinazoline derivatives [23]–[25]. Altogether, these considerations led us to investigate whether the anti-HCV activity displayed by these compounds might be due to inhibition of a cellular kinase.

Recently, several small-interfering RNA (siRNA) screening campaigns have identified type III phosphatidylinositol 4-kinases (PI4K) as crucial host factors for HCV replication. In particular, PI4KIIIα was found to be required for HCV RNA replication in a cell line- and genotype-independent manner, whereas the requirement for the β isoform was observed to be less dramatic and limited to Con-1 (genotype 1b) replicons [26]–[29]. It was shown that the catalytic activity of PI4KIIIα is required to rescue HCV replication in cells with a stable knock-down of PI4KIIIα. In addition, it has been proposed that NS5A stimulates PI4KIIIα activity by direct interaction via domain I [30]–[32]. All these observations taken together made us consider the phosphatidylinositol 4-kinases a potential alternative candidate target for 4-anilino quinazoline inhibitors of HCV replication.

In this paper, we present evidence that AL-9, a member of this class of compounds previously reported to target NS5A, inhibits PI4P formation by direct inhibition of phosphatidylinositol 4-kinase IIIα (PI4KIIIα). In addition, we provide evidence that pharmacological inhibition of PI4KIIIα with AL-9 results in altered subcellular distribution of NS5A similar to that observed after RNAi knock-down of the PI4KIIIα mRNA, strongly supporting a mechanism of HCV inhibition mediated by the inhibition of PI4KIIIα. Moreover, we show that HCV subverts components of the phosphatidylinositol-4 phosphate (PI4P) pathway to function in favor of its own life cycle, thereby enriching the PI4P concentration in the membranous web while depleting the plasma membrane PI4P pool.

## Results

### Compound AL-9 inhibits HCV replication in vitro

**Figure 1:**
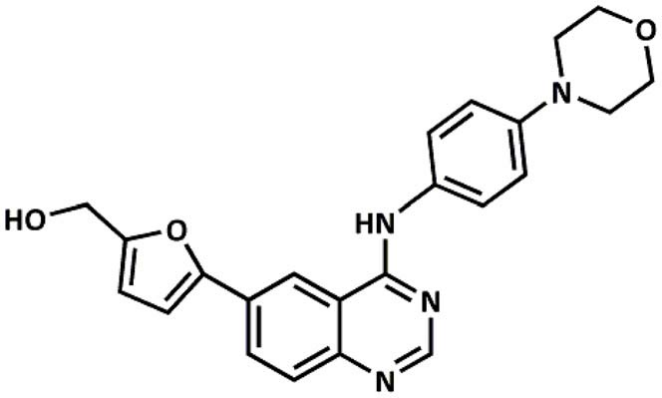
Chemical structure of AL-9. For the synthetic pathway and procedure see Supporting Information.

AL-9 is a member of 4-anilino quinazoline-containing HCV replication inhibitors described previously ([21]; Figure 1). In order to confirm its anti-HCV activity, we tested the effect of this compound on HCV replication in Huh7.5 cells stably expressing genotype 1b or 2a subgenomic replicons (Con1-SR and JFH-A4, respectively). The EC_50_ values, calculated by measuring viral RNA after incubation with AL-9 for three days, are reported in Table 1. Replicon EC_50_ values for AL-9 were found to be 0.29 µM and 0.75 µM for genotype 1b and 2a, respectively. In order to prove that AL-9 inhibits HCV replication not only in the context of a HCV subgenomic replicon, but also in the context of the complete viral life-cycle, we determined the inhibitory activity using the J6/JFH-1 HCV virus. In this case, the EC_50_ value was found to be 1.2 µM, a figure comparable with the result obtained with genotype 2a subgenomic replicon. CC_50_ values are shown for Con1-SR, JFH-A4 and Huh7.5 cells, respectively. In summary, AL-9 inhibits HCV across different genotypes with activity in the sub-micromolar to low micromolar range in the absence of significant cytotoxic effects.

**Table 1:** List of EC_50_ values of AL-9 for different HCV genotypes.

	genotype	EC_50_ (µM)	CC_50_ (µM)
Con1-SR	1b	0.29 (+/−0.09)	29.3 (+/−2.8)
JFH-A4	2a	0.75 (+/−0.15)	18.9 (+/−3.2)
Huh7.5+J6/JFH-1 HCV	2a	1.2 (+/−0.37)	25.1 (+/−4.6)*

Huh7.5 cells replicating subgenomic replicons of genotype 1b or 2a (Con1-SR and JFH-A4, respectively) or Huh7.5 cells infected with the chimeric virus J6/JFH were treated with AL-9 for three days and intracellular viral RNA was measured by real time PCR. The data are representative of at least three independent experiments, and the standard deviations are shown.

*: CC_50_ measured in uninfected Huh7.5 cells.

### AL-9 is an inhibitor of PI4KIIIα

**Figure 2:**
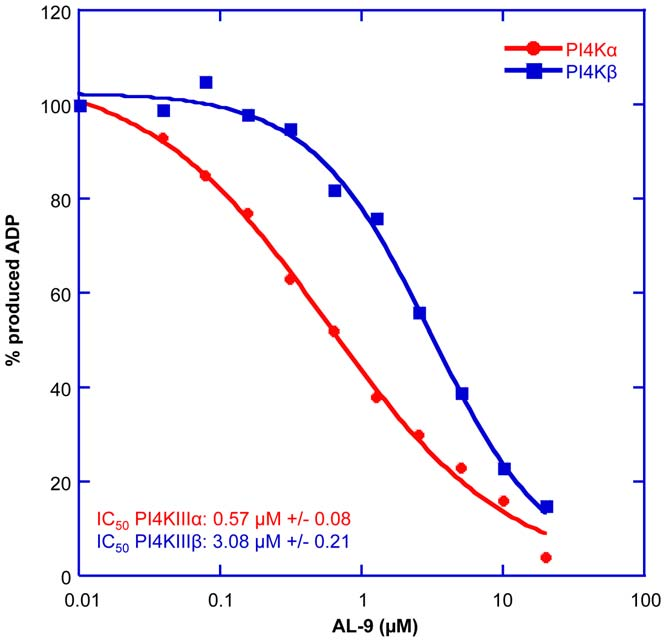
Inhibitory dose-response curve of AL-9 for purified PI4KIIIα and PI4KIIIβ. The enzymes were preincubated for 10 min with the indicated concentrations of AL-9 or DMSO and the reaction was started by addition of 100 µM ATP and 150 µM PI∶PS substrate as described in Materials and Methods. Activity, measured as conversion of ATP to ADP, is expressed as percent of the DMSO control. Shown is a representative experiment of three independent experiments performed in duplicate. IC_50_ and SD of PI4KIIIα and PI4KIIIβ are indicated.

In the following experiment, we investigated whether AL-9 inhibits the purified type III phosphatidylinositol 4-kinases PI4KIIIα and PI4KIIIβ (Figure 2). Both enzymes were inhibited by AL-9 with a five-fold preference for PI4KIIIα (IC_50_ of 0.57 µM and 3.08 µM, respectively). This result demonstrates that AL-9 inhibits type III PI4 kinases *in vitro* at concentrations similar to those required for its anti-HCV activity, displaying a moderate selectivity for the α over the β isoform. We also tested the activity of AL-9 on two class I PI3-kinases (p110α and p110β). While PI3-kinase p110α was inhibited with an IC_50_ of 1.1 µM, the potency of AL-9 for PI3-kinase p110β was significantly lower (40% inhibition @10 µM, data not shown).

Our hypothesis is that AL-9 inhibits HCV replication *via* inhibition of PI4KIIIα. Thus, we wanted to assess whether AL-9 also inhibited PI4KIIIα in living cells. To this aim, we needed to set up an assay that allowed us to monitor the activity of this kinase in intact cells. PI4KIIIα is primarily localized to the ER, whereas PI4KIIIβ is localized to the Golgi membranes [33]. It was shown that PI4KIIIβ contributes to the synthesis of PI4P at the Golgi membranes [34], [35]. Subcellular localization of the enzymes, however, does not always coincide with their function. Thus, PI4KIIIα, considered to be an ER-resident enzyme, has previously been shown to be critical for the generation and maintenance of the plasma membrane PI4P pool during phospholipase C activation and Ca_2_ signaling in HEK-293 or Cos-7 cells [35], [36] as well as in resting Cos-7 cells [37]. Whether PI4KIIIα is responsible for the maintenance of the plasma membrane PI4P pool under normal cell culture conditions in hepatoma cells is currently not known. Hammond et al [37] have developed immunocytochemical techniques that enable selective staining of the PI4P pool present in the plasma membrane (plasma membrane staining protocol) or in the intracellular membranes (Golgi staining protocol), respectively. We used this technique, in combination with RNA gene silencing or pharmacological inhibition, to decipher which of the type III enzymes participates in the synthesis of the Golgi- or plasma membrane PI4P-pools in Huh7.5 hepatoma cells.

**Figure 3:**
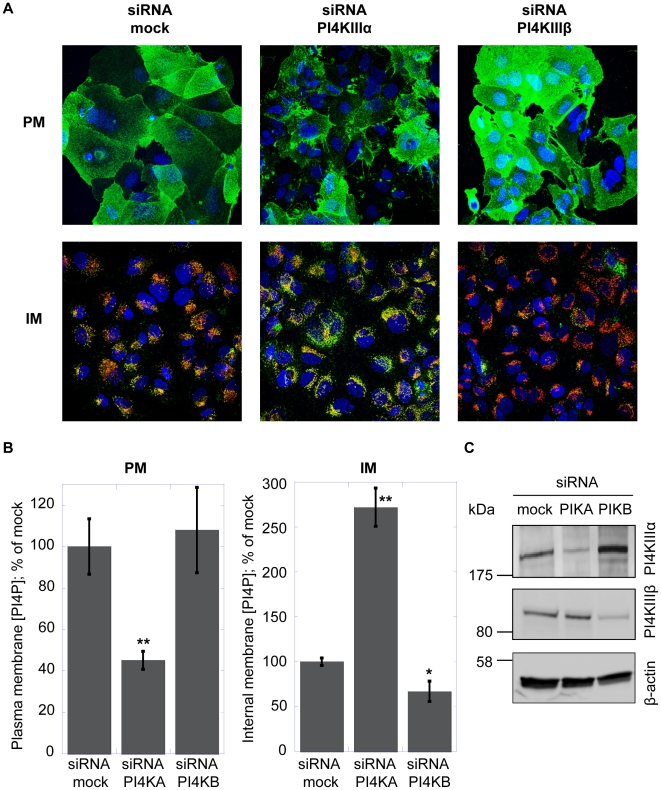
RNA interference analysis of PI4P production in Huh7.5 cells. Huh7.5 cells were treated with irrelevant (mock) siRNA or siRNA targeting PI4KIIIα or PI4KIIIβ as detailed in Materials and Methods. The data were collected three days after initial siRNA transfection. (A) Confocal microscopy images of Huh7.5 cells treated with PI4KIIIα siRNA, PI4KIIIβ siRNA or mock siRNA. Cells were fixed and stained as described in Materials and Methods. PI4P (green) localized to the plasma membrane (PM) was detected using the plasma membrane staining protocol (upper panel) [37]. Nuclei were stained by the Hoechst dye (blue). PI4P in the intracellular membranes (IM) was revealed using the Golgi staining protocol (lower panel). Together with PI4P, Golgi membranes were stained with the Golgi marker giantin (red). Colocalization of PI4P with Golgi membranes results in yellow color. (B) Quantification of PI4P levels by immunofluorescence analysis. Changes in mean fluorescence intensity relative to the control (mock siRNA) are shown. Four randomly picked fields were analyzed per each condition, as described in Materials and Methods. Data are presented as averages ± SEM. *, p<0.05; **, p<0.01. (C) Immunoblot analysis of protein expression after RNAi silencing. Lysates prepared from Huh7.5 cells transfected with irrelevant siRNA (mock), PI4KIIIα siRNA (PIKA) or PI4KIIIβ siRNA (PIKB) were analyzed by immunoblotting with PI4KIIIα, PI4KIIIβ or β-actin antibodies as indicated in the figure. Positions of the protein molecular weight markers are shown on the left side.

To address which type III PI4 kinase is responsible for the synthesis of the different cellular PI4P pools, Huh7.5 cells were treated with siRNAs targeting PI4KIIIα, PI4KIIIβ or an unrelated siRNA (mock-siRNA) as described in the Materials and Methods section. Immunoblots assays show specific knockdown of PI4KIIIα or PI4KIIIβ by their corresponding siRNAs (Figure 3C). Three days after siRNA treatment, PI4P was revealed either by the plasma membrane staining protocol (Figure 3A, upper panel) or by the Golgi membrane staining protocol (Figure 3A, lower panel). In cells treated with the unrelated siRNA (mock-siRNA), PI4P was detected both in the plasma membrane and in intracellular membranes. Intracellular PI4P was localized primarily in the Golgi membranes, as judged by the colocalization with the Golgi marker giantin. Silencing of PI4KIIIα resulted in a significant decrease of the PI4P level in the plasma membrane. Concomitantly with the decrease in the plasma membrane PI4P levels, we consistently observed a pronounced increase of PI4P level in the Golgi membrane following PI4KIIIα knockdown. In the case of PI4KIIIβ knockdown, we observed a ∼30% decrease of Golgi membrane PI4P level, whereas the PI4P levels of the plasma membrane remained substantially unaffected (Figure 3B).

These results are in line with the previously reported role for PI4KIIIα in maintaining the PI4P plasma membrane pool [35]–[37] and confirm the importance of PI4KIIIβ for the synthesis of at least part of the Golgi membrane PI4P [34], [35]. We also observed that decreased expression of PI4KIIIα resulted in an unexpected increase in the level of the Golgi membrane pool (Figure 3A), suggesting a complex level of cross-talk between the cellular type III PI4 kinases in maintaining the physiological PI4P levels at the Golgi membrane, at least in our experimental model.

**Figure 4 ppat-1002576-g004:**
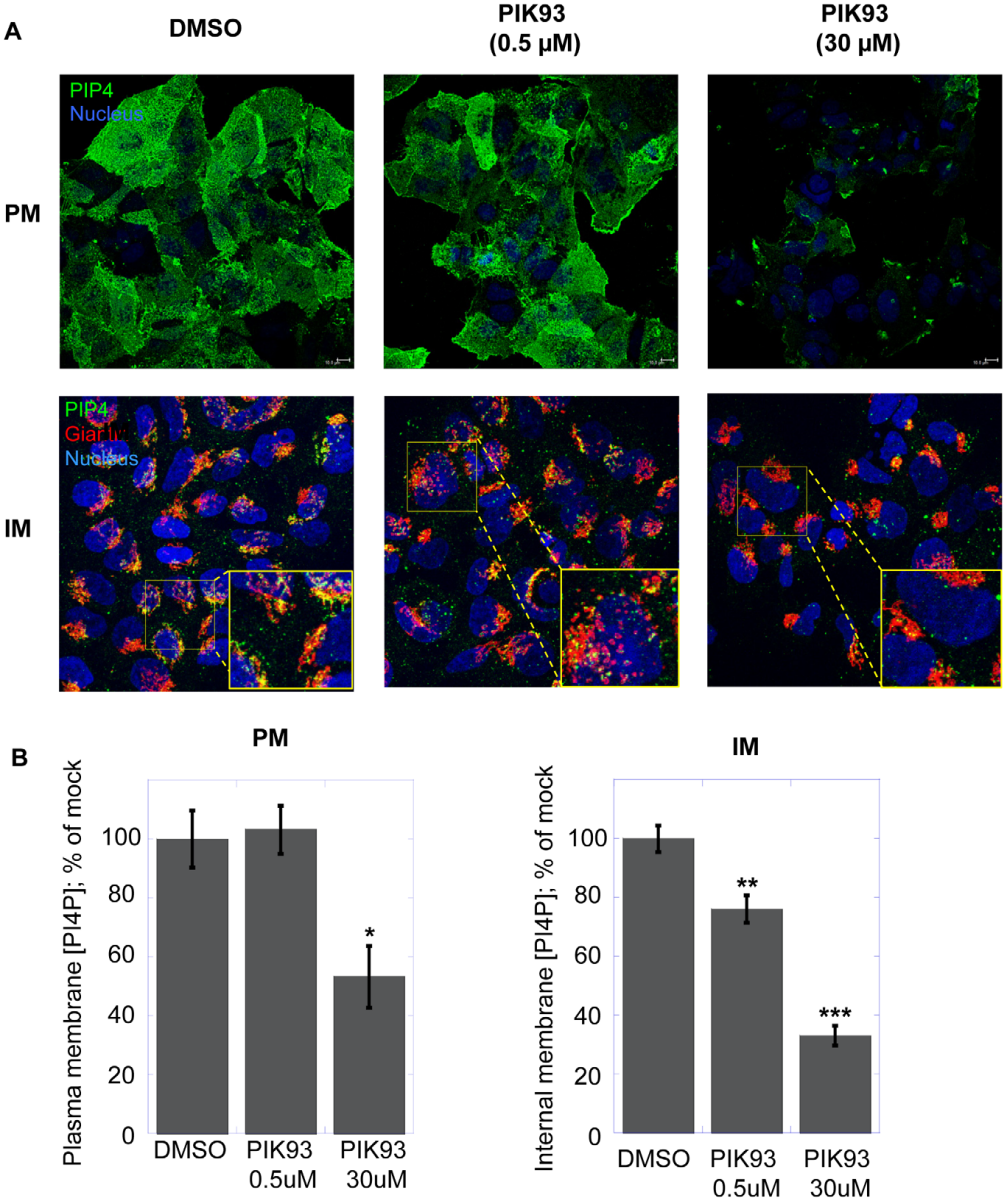
Effect of PIK93 on Golgi or plasma membrane PI4P in Huh7.5 cells. (A) Confocal microscopy images of Huh7.5 cells incubated with DMSO (left column), 0.5 µM PIK93 (central column) or with 30 µM PIK93 (right column) for 2 hours prior to fixation and staining as described in Materials and Methods. PI4P (green) localized to the plasma membrane (PM) was detected using the plasma membrane staining protocol (upper panel) [37]. Nuclei were stained by the Hoechst dye (blue). PI4P in the intracellular membranes (IM) was revealed using the Golgi staining protocol (lower panel). Together with PI4P, Golgi membranes were stained with the Golgi marker giantin (red). Colocalization of PI4P with Golgi membranes results in yellow color (zoomed sections are indicated by a yellow square). (B) Quantification of PI4P levels by immunofluorescence analysis. Changes in mean fluorescence intensity relative to the control (DMSO) are shown. Four randomly picked fields were analyzed per each condition. Normalization was performed as detailed in Materials and Methods. Data are presented as averages ± SEM. *, p<0.05; **, p<0.01; ***, p<0.001.

In order to confirm and extend the results described above, we utilized a known pharmacological inhibitor of the type III PI4 kinases. PIK93 was previously exploited to distinguish between the roles of the two PI4KIII isoforms [38], [39]. In particular, a concentration of 0.5 µM PIK93 is expected to affect only PI4KIIIβ, whereas 30 µM PIK93 should inhibit both PI4KIIIβ and PI4KIIIα. Thus, Huh7.5 cells were treated with 0.5 µM or 30 µM PIK93 or with DMSO as control. After two hours of incubation, PI4P was revealed either by the plasma membrane staining protocol (Figure 4A, upper panel) or by the Golgi staining protocol (Figure 4A, lower panel). PI4P levels associated with the Golgi membranes decreased by ∼25% after incubation with 0.5 µM PIK93 (Figure 4B). This is in line with PI4KIIIβ contributing to the production of PI4P present in the Golgi membranes (PI4KIIα, another contributor of Golgi-localized PI4P is not inhibited by PIK93 [38], [39]). Increasing PIK93 concentration to 30 µM further increased the inhibition of the intracellular membrane PI4P pool, to ∼65% (Figure 4B). This could be due to a more complete inhibition of PI4KIIIβ; however, based on this experiment, we cannot rule out a contribution of PI4KIIIα activity to the maintenance of the Golgi membrane PI4P pool. In contrast to what observed in the Golgi-associated membranes, the plasma membrane PI4P level was not significantly affected upon incubation with 0.5 µM PIK93, but decreased by nearly 50% after incubation with 30 µM of PIK93 (Figure 4B). Combined with the RNAi experiments described above, these results support the notion that, in Huh7.5 cells, PI4KIIIα is involved in the maintenance of the plasma membrane PI4P pool, whereas PI4KIIIβ is at least partly responsible for the maintenance of the Golgi membrane PI4P pool.

**Figure 5:**
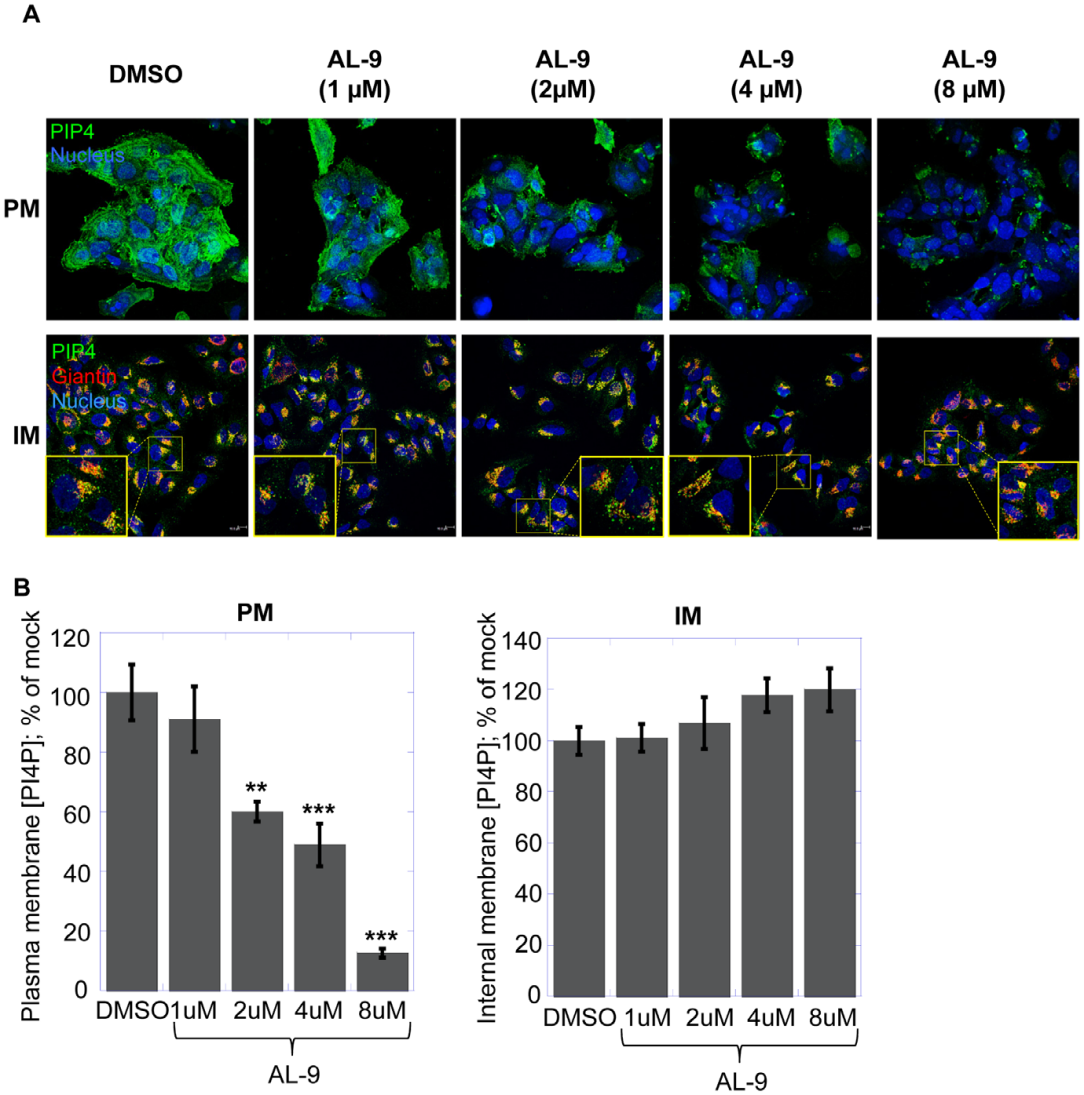
AL-9 inhibits PI4KIIIα in Huh7.5 cells. (A) Confocal microscopy images of Huh7.5 cells treated for 2 hours with DMSO (left column) or with 1, 2, 4 or 8 µM of AL-9 (columns 2 to 5). PI4P (green) localized to the plasma membrane (PM) was detected using the plasma membrane staining protocol (upper panel) [37]. Nuclei were stained by the Hoechst dye (blue). PI4P in the intracellular membranes (IM) was revealed using the Golgi staining protocol (lower panel). Together with PI4P, Golgi membranes were stained with the Golgi marker giantin (red). Colocalization of PI4P with Golgi membranes results in yellow color (zoomed sections are indicated by a yellow square). (B) Quantification of PI4P levels by immunofluorescence analysis. Changes in mean fluorescence intensity relative to the control (DMSO) are shown. Four randomly picked fields were analyzed per each condition. Normalization was performed as detailed in the Materials and Methods. Data are presented as averages ± SEM. **, p<0.01; ***, p<0.001.

We then evaluated the PI4K inhibitory activity of AL-9 in Huh7.5 cells using the same methodology. Briefly, Huh7.5 cells were incubated either with DMSO or with increasing concentration of AL-9 (1, 2, 4 or 8 µM) for two hours (Figure 5A). Treatment with AL-9 gradually reduced the amount of PI4P in the plasma membrane (Figure 5B). Conversely, the concentration of PI4P in the Golgi-associated membranes remained substantially unaltered up to the highest AL-9 concentration used (Figure 5B).

In all, the results described above suggest that AL-9 inhibits PI4KIIIα also in living cells, while not appreciably affecting the activity of PI4KIIIβ. This is in line with the selectivity for PI4KIIIα over PI4KIIIβ observed in the biochemical assays.

### HCV alters the intracellular and plasma membrane distribution of PI4P

Viral infection induces modification of intracellular membrane structures [40] and, for some RNA viruses including HCV, it has been shown that these induced membranous structures are highly enriched for PI4P [32], [41]. Before testing the activity of AL-9 in HCV-infected cells, we wanted to know what the impact of HCV on cellular membrane structures was, with special regard to the subcellular membrane distribution of PI4P.

**Figure 6:**
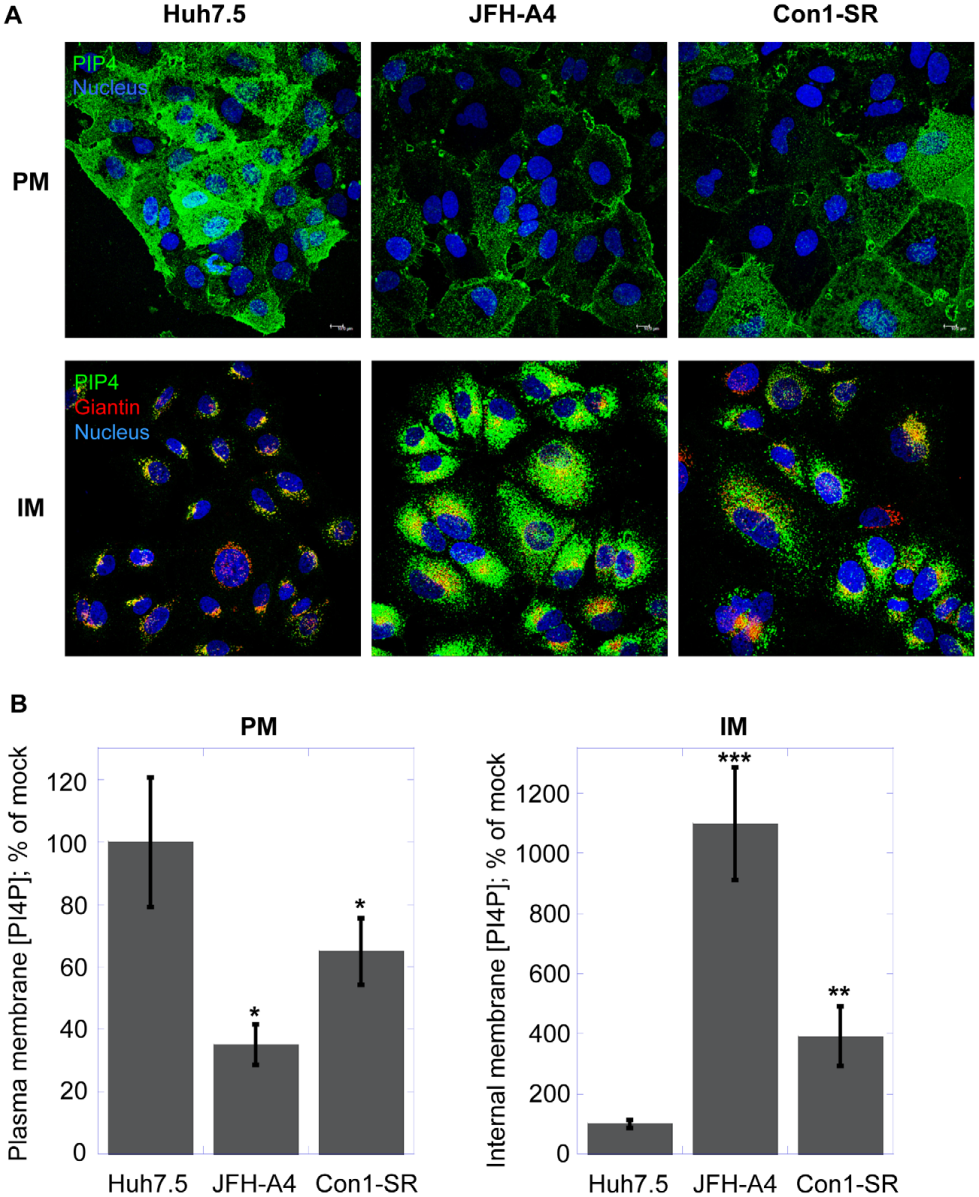
HCV impacts subcellular PI4P distribution. (A) Huh7.5 cells, JFH-A4 and Con1-SR cells were analyzed by confocal microscopy for the presence of PI4P (green) in the plasma membranes (PM, upper panel) or in the intracellular membrane (IM, lower panel) using the protocols described in Materials and Methods. Nuclei were stained by the Hoechst dye (blue). For internal membrane staining, giantin (red) was used as a specific marker for Golgi membranes. (B) Quantification of PI4P levels by immunofluorescence analysis. Changes in mean fluorescence intensity relative to the control (Huh7.5 cells) are shown. Four randomly picked fields were analyzed per each condition. Normalization was performed as detailed in Materials and Methods. Data are presented as averages ± SEM. *, p<0.05; **, p<0.01; ***, p<0.001.

Naïve Huh7.5 cells or cells actively replicating the genotype 2a or 1b HCV subgenomic replicon were investigated for their PI4P concentration in internal membranes or plasma membranes, respectively (Figure 6A). As previously shown, cells expressing the HCV replicon form a membranous web that is highly enriched for PI4P (Figure 6A, lower panel). The level of PI4P in these virus-specific membrane structures is markedly higher in JFH-A4 cells, containing the very efficient genotype 2a JFH-1 replicon, compared to the Con1-SR cells, which are based on the genotype 1b Con1 replicon, possibly mirroring the different RNA replication efficiency. It is well established that the kinase responsible for the production of the PI4P pool present in these structures is PI4KIIIα. In the current model, PI4KIIIα interacts with the viral protein NS5A, leading to up-regulation of the kinase activity and accumulation of PI4P in the virus-specific membranous web [30]–[32]. Conversely, the results shown in Figures 3–5 suggest that – in absence of viral replication – a major function of PI4KIIIα is the synthesis of the PI4P pool in the plasma membrane. We therefore asked ourselves whether the presence of HCV could not only influence distribution and enrichment of PI4P in internal membranes, but also alter the PI4P plasma membrane pool. In Figure 6A, we show that, concomitantly with the increase of PI4P in the internal membranes (lower panel), HCV replication promotes a marked decrease of PI4P concentration in the plasma membrane (upper panel). Relative quantification of the PI4P levels in the different experimental conditions is shown in Figure 6B. This experiment demonstrates that the presence of HCV causes a dramatic change of PI4P localization in cellular membranes, whereby the increase of PI4P concentration in the virus-specific membranous structures appears to be accompanied by a depletion of the PI4P pool normally present in the plasma membrane.

**Figure 7:**
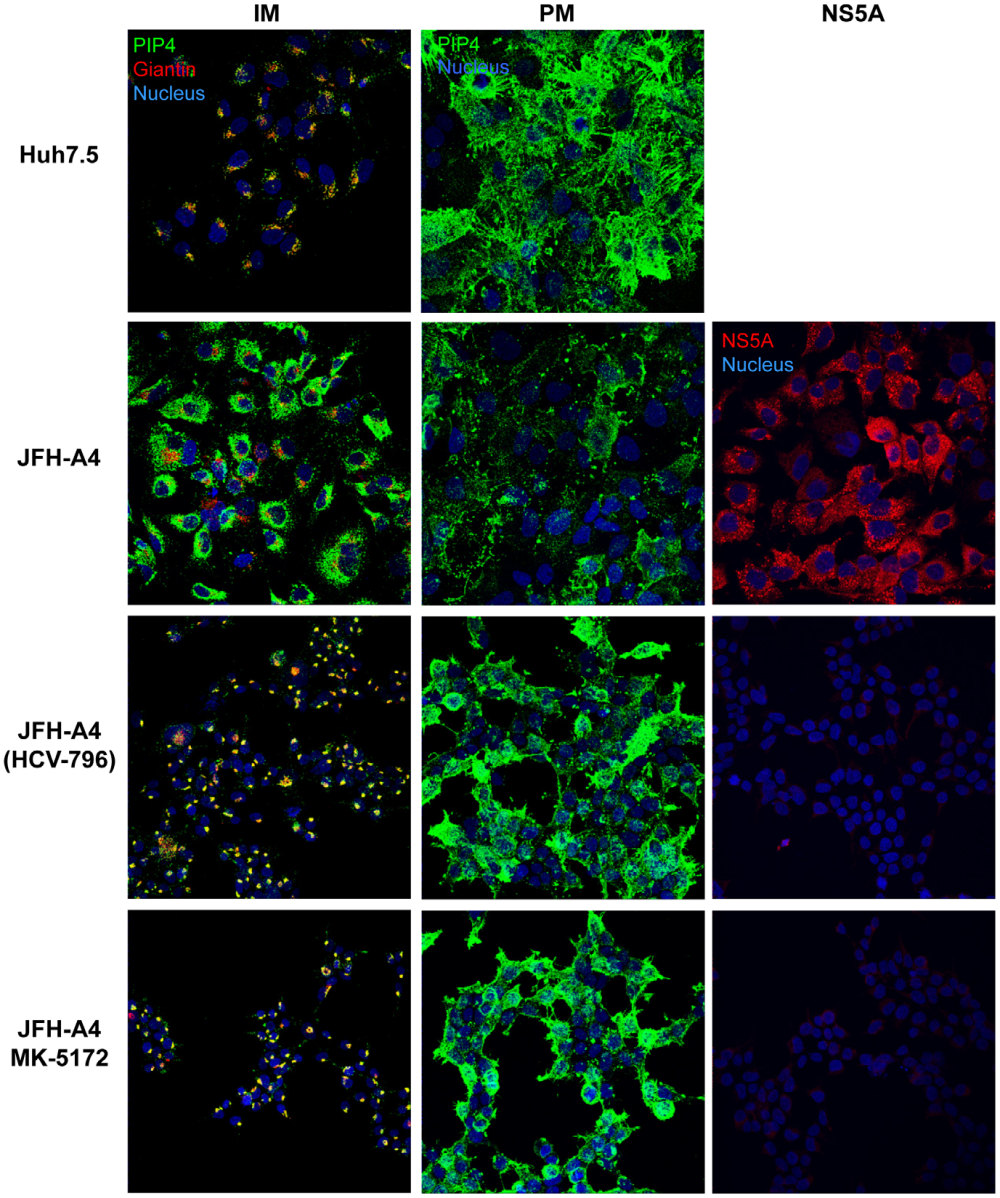
Reversibility of HCV-induced changes in PI4P subcellular distribution. JFH-A4 cells were incubated for 14 days with the HCV RdRP inhibitor HCV-796 (2 µM) or the HCV NS3/4A protease inhibitor MK-5172 (0.2 µM). Cure from HCV was controlled by detection of NS5A with a specific NS5A antibody (red, right column). As control, untreated Huh7.5 cells or JFH-A4 cells were used. Cells were fixed and PI4P (green) was detected in the internal membranes (IM, left column) or in the plasma membrane (PM, central column). For internal membrane staining giantin (red) was used as a specific marker for Golgi membranes. Nuclei were stained by the Hoechst dye (blue).

We next investigated whether HCV-associated changes in PI4P distribution could be reverted upon cure of the HCV replicon by specific inhibitors. We treated JFH-A4 cells for two weeks either with the HCV RdRP inhibitor HCV-796 or with the HCV NS3/4A protease inhibitor MK-5172 and followed PI4P localization in internal membranes and in the plasma membrane (Figure 7). Independent of the type of inhibitor used, the result shows that the HCV-induced PI4P-enriched membranous web in JFH-A4 cells disappeared upon suppression of HCV replication and that the intracellular PI4P localization returned to the Golgi-localization as observed in the naïve Huh7.5 cells (left column). In parallel, the plasma membrane concentration of PI4P increases to the levels observed in naïve cells (middle column). NS5A staining (right column) as well as real-time RT-PCR (not shown) indicated that the prolonged treatment with HCV-inhibitor led to complete and stable suppression of viral protein expression and undetectable level of HCV RNA. Thus, removal of HCV RNA brings PI4P synthesis and distribution back to a level comparable to naïve Huh7.5 cells. It is worth of note, however, that the previous presence of HCV replicons in the cured cells induced some irreversible morphological changes of unknown nature, such as a smaller cell size.

### AL-9 inhibits PI4KIIIα in HCV-replicating cells

**Figure 8:**
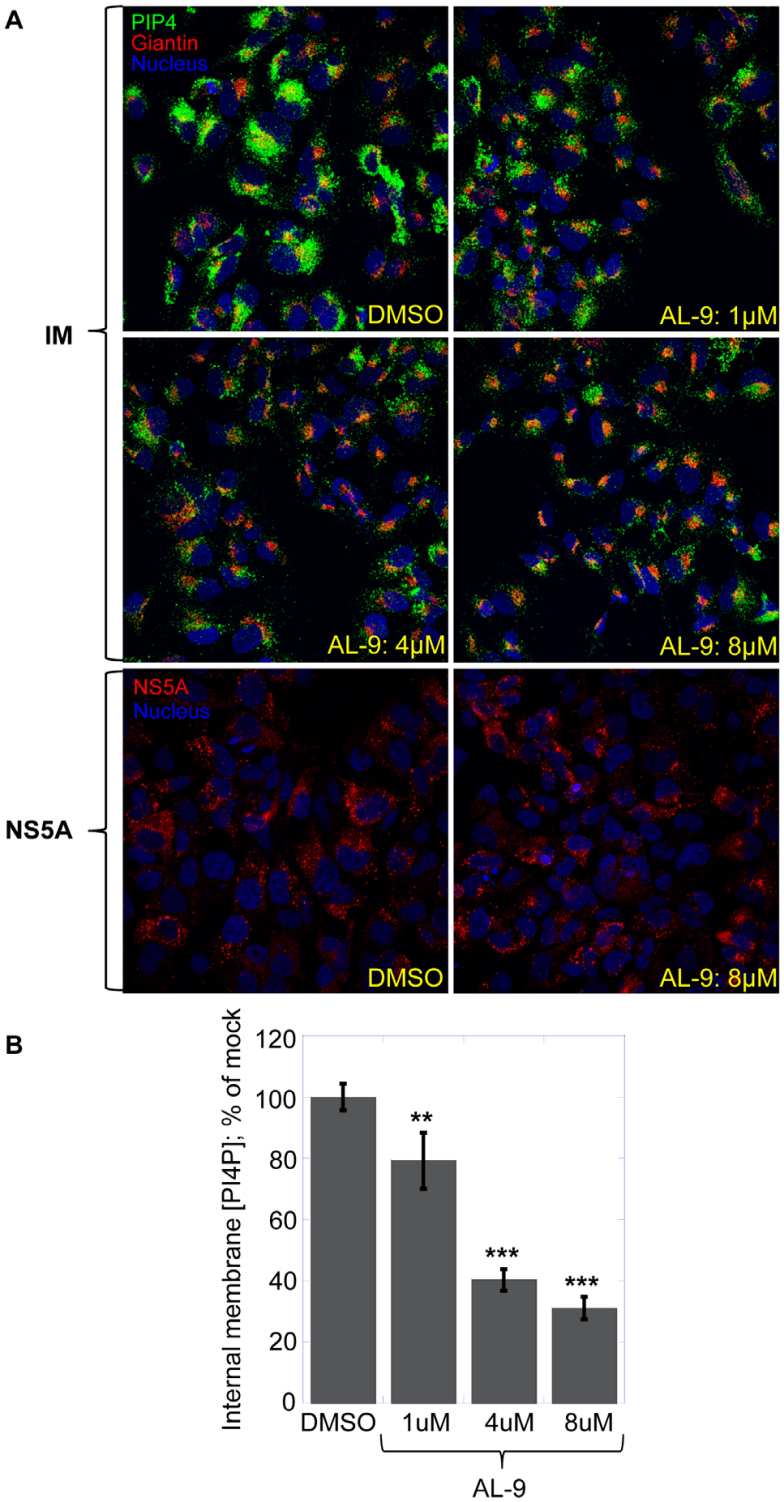
AL-9 inhibits PI4KIIIα in HCV-replicating cells. (A) JFH-A4 cells were treated with DMSO or AL-9 for 4 hours and internal membranes were stained for PI4P (green) and the Golgi marker giantin (red) using the Golgi staining protocol, as described in Materials and Methods. DMSO or AL-9 concentrations are indicated within the image. Alternatively, cells were stained for NS5A as described in Materials and Methods (indicated as NS5A). Nuclear DNA was stained with Hoechst dye (blue). PI4KIIIα, associated with the HCV-associated membranous web is inhibited by AL-9. The decrease of PI4P is not due to inhibition of the HCV replication indicated by unchanged NS5A expression and localization (lower panel). (B) Quantification of PI4P levels by immunofluorescence analysis. Changes in mean fluorescence intensity relative to the control (DMSO) are shown. Four randomly picked fields were analyzed per each condition. Normalization was performed as detailed in Materials and Methods. Data are presented as averages ± SEM. **, p<0.01; ***, p<0.001.

We have shown that PI4KIIIα is inhibited by AL-9 in naïve Huh7.5 cells. As discussed above, in HCV-replicating cells, the kinase activity of PI4KIIIα is up-regulated by a direct protein-protein interaction with the viral protein NS5A [30], [32]. In the following experiment (Figure 8), we explored whether AL-9 is able to inhibit PI4KIIIα also in this context. JFH-A4 cells were incubated with increasing concentration of AL-9 for 4 hours and PI4P concentration in the HCV membranous web was followed by immunostaining (Golgi staining protocol). Treatment of cells with AL-9 lead to clear inhibition of PI4P accumulation in the HCV membranous web. Incubation with 8 µM AL-9 depleted as much as 70% of the PI4P present in the intracellular membranes of replicon-containing cells. This result confirms that AL-9 inhibits PI4KIIIα independent of its membranous localization and suggests that this inhibition could be responsible for the observed antiviral effect. Since AL-9 has anti-HCV activity in the concentration range used here, longer incubation of HCV replicons with AL-9 results in inhibition of HCV RNA- and protein-synthesis. As a consequence, the PI4P-enriched HCV membranous web would disintegrate. In this case loss of PI4P in the internal membranes could be not a direct consequence of PI4KIIIα inhibition, but a consequence of disintegration of the HCV membranous web.

In order to rule out this possibility, we checked localization of NS5A, a presumed marker for HCV replication sites, after 4 hours of incubation with AL-9. Localization of NS5A does not change, suggesting that AL-9 does not significantly change the structure of the HCV membranous web upon 4 hours of treatment. Moreover, incubating the same replicon cells for 4 hours with HCV-796, an HCV polymerase inhibitor, did not lead to appreciable depletion of the membranous web PI4P pool indicating that the loss of PI4P in the HCV-induced intracellular membranes is the direct consequence of inhibition of PI4KIIIα, and not the consequence of inhibition of HCV replication. Additional evidence is provided in the experiment below, in which expression of the HCV polyprotein, and consequently formation of a membranous web, was driven by cDNA plasmid rather than by autonomously replicating HCV RNA.

### PI4KIIIα inhibition by AL-9 alters sub-cellular distribution of NS5A

**Figure 9:**
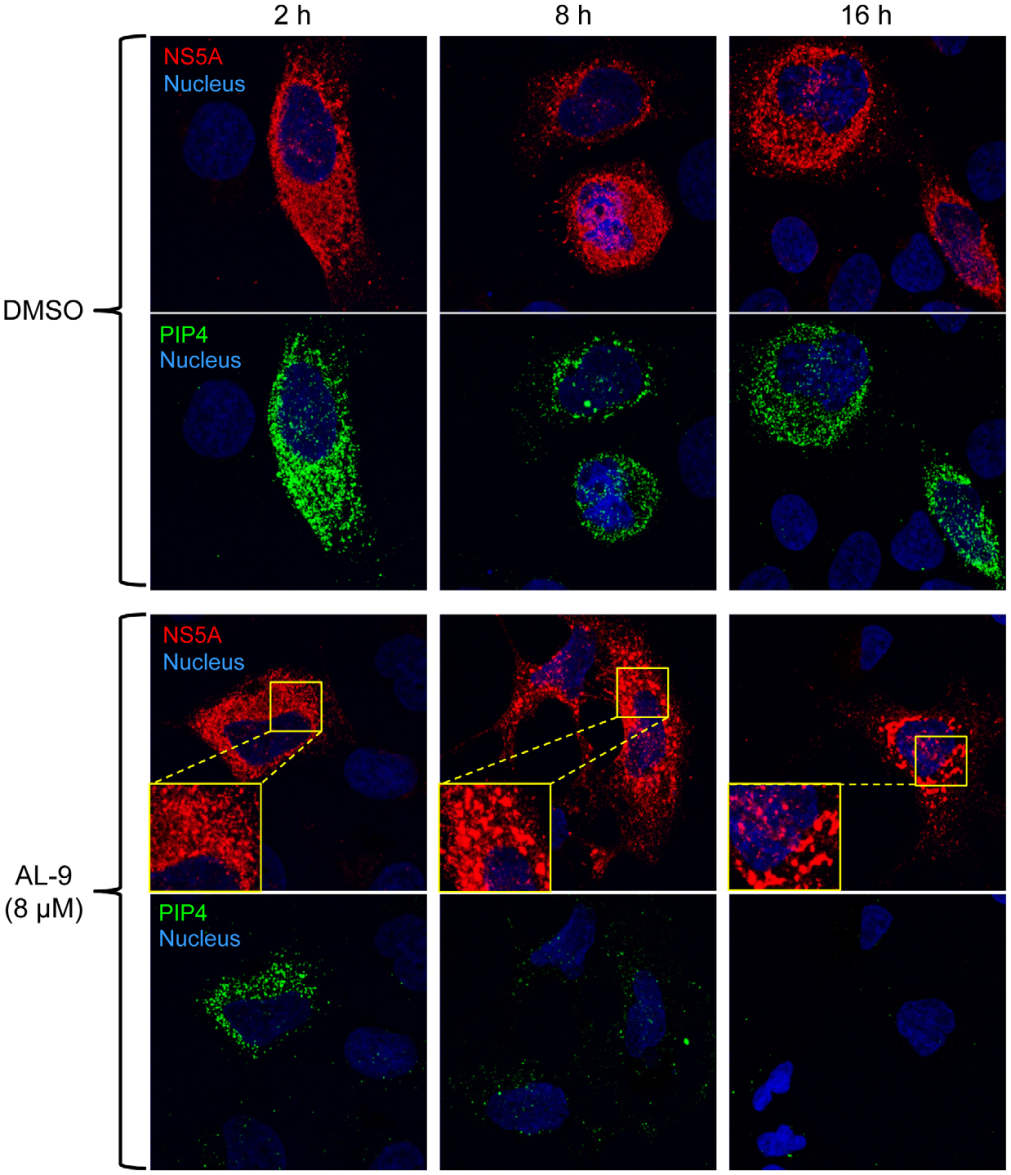
Inhibition of PI4KIIIα by AL-9 induces the formation of large NS5A clusters. Huh7-Lunet/T7 cells were transiently transfected with the plasmid pTM-NS3-5B which expresses the HCV nonstructural proteins under the control of the T7 RNA polymerase promoter. Cells were treated with DMSO (upper panels) or 8 µM AL-9 (lower panels) for 2, 8 or 16 hours and were then stained for NS5A (red) and PI4P (green) using the Golgi staining protocol as described in Materials and Methods. Nuclear DNA was stained with the Hoechst dye (blue). Zoomed sections are indicated by a yellow square. Long incubation with AL-9 (8–16 hours) results in increased NS5A clustering and concomitantly a decrease of PI4P in the internal membranes.

It was previously shown that knock-down of PI4KIIIα expression by RNAi resulted in the production of large NS5A clusters. This was achieved in an experimental setting where the HCV polyprotein was expressed from DNA constructs, thus avoiding potential confounding effects due to inhibition of HCV RNA replication [27], [32]. We wanted to assess whether pharmacological inhibition of PI4KIIIα kinase activity would lead to similar effects on NS5A subcellular localization. Thus we followed the effect of AL-9 on NS5A localization after transient DNA transfection in Huh7-Lunet/T7 cells with a plasmid expressing genotype 2a nonstructural proteins NS3-NS5B under the control of a T7 promoter [42]. Cells were treated either with DMSO (upper panels) or with 8 µM AL-9 (lower panels) for 2, 8 or 16 hours and localization of NS5A as well as PI4P were followed by indirect fluorescence microscopy (Figure 9). Cells successfully transfected with the HCV polyprotein expressed NS5A and induced the PI4P-enriched membranous web. After 8 hours of treatment with AL-9, changes in NS5A localization in form of larger clusters become visible. At the same time, PI4P concentration in the membranous web started to decrease. After 16 hours of incubation with AL-9, NS5A was concentrated almost exclusively in large clusters. At this time-point, PI4P in the internal membranes had completely vanished.

In summary, this experiment shows that, in cells expressing the HCV polyprotein from cDNA, prolonged treatment with AL-9 results in a redistribution of NS5A into large clustered structures with high resemblance to the structures previously observed after silencing of the PI4KIIIα gene by RNAi [27], [32]. Concomitantly, we observed a depletion of the PI4P pool present in the HCV-induced membranous structures. These results indicate that the catalytic activity of PI4KIIIα is directly or indirectly required for the proper localization of HCV NS5A protein into the membranous web. Furthermore, the experiment just described lands additional support to the notion that the antiviral effect of AL-9 is mediated by the inhibition of PI4KIIIα.

## Discussion

In the present paper, we show that a compound belonging to the class of 4-anilino quinazoline inhibitors of HCV replication is an inhibitor of PI4KIIIα, a cellular lipid kinase required for viral replication.

PI4KIIIα belongs to the family of type III phosphatidylinositol 4-kinases, enzymes that catalyze the conversion of phosphatidylinositol to phosphatidylinositol 4-phosphate (PI4P). PI4P is the most abundant monophosphorylated inositol phospholipid in mammalian cells and the importance of this phospholipid is just started to be unraveled [43]. In addition to playing important roles in intracellular signaling and membrane trafficking, phosphatidylinositol lipids and their metabolizing enzymes are also exploited by many different viruses in order to transform cellular membranes in structures supporting their replication [40], [44], [45]. PI4KIIIβ was shown to be a host factor required for enterovirus replication [41], whereas several reports have demonstrated that PI4KIIIα is crucial for HCV replication [26]–[29]. Owing to the importance of this pathway, the need for specific inhibitors of PI4III kinases is increasing. Only recently, some enviroxime-like compounds with antiviral activity against enterovirus have been demonstrated to target PI4KIIIβ. One of these agents is a very specific inhibitor of the β-isoform of the type III PI4-kinases [46]. So far, no such compound exists for the PI4KIII-α isoform. A commonly used inhibitor for type III phosphatidylinositol 4-kinases is PIK93, which has originally been designed to inhibit class I PI3-kinases [38]. This compound allows differential inhibition of PI4KIIIβ alone or PI4KIIIα and PI4KIIIβ together depending on the concentration used. In this paper, we show that a 4-anilino quinazoline derivative, termed AL-9 (Figure 1 and Figure S1), is able to inhibit PI4KIIIα in a test tube as well as in living cells. AL-9 inhibited purified PI4KIIIα, with a moderate (∼5-fold) selectivity over the β isoform (Figure 2). In cell culture, we observed that treatment with AL-9 efficiently inhibits the maintenance of the plasma membrane PI4P pool in Huh7.5 cells while not significantly affecting the Golgi membrane pool at the highest concentration used (Figure 5). This finding is in line with the moderate selectivity observed in the biochemical assay. Thus, AL-9 represents a lead candidate for the development of more potent and more specific inhibitors of PI4KIIIα.

Anti-HCV compounds of the 4-anilino quinazoline class were previously assumed to exert their antiviral effect via inhibition of the viral protein NS5A. This conclusion rested on analysis of the mutations found in the HCV replicon in association with resistance to these agents [21]. Mutations generated against 4-anilino quinazolines were localized mainly in NS5A, in triplets that occurred all in NS5A or appeared concomitant with changed in NS4B or NS5B [17], [22] (see also Introduction). Reverse genetic experiments, in which these mutations were reintroduced in the replicon (single, double and triple combinations), however, did not support a role for these mutations in conferring resistance to 4-anilino quinazolines [17]. In order to assess whether the reported mutations conferred any level of resistance to AL-9, we independently performed reverse genetics studies in which selected mutations triplets, reported to be associated with the higher level of resistance, were reintroduced in a genotype 1b replicon with the same genetic background as the one reported in the original resistance study (Figure S2). These mutation triplets are: FAG: L199F+V362A+S390G (NS5A), DLD: E212D+P299L+V388D (NS5A), and PPA: T200P+S370P(NS5A)+S76A(NS5B). We observed that the replicon containing the first triplet lost the ability to replicate at significant level. For replicons containing the latter two combinations of mutations, RNA replication could be measured, although at a lower level compared to the parental construct (35% and 20%, respectively). These replicons, however, remained equally sensitive to AL-9 as the parental replicon (Figure S2), opening the question as to which really is the target of this compound class. We are currently trying to select HCV replicons resistant to AL-9. So far we were unable to identify mutations that confer resistance to AL-9.

Our new data on AL-9 suggest that inhibition of HCV replication by 4-anilino quinazoline compounds is a consequence of PI4KIIIα inhibition. Our conclusion rests on a number of experimental findings. First of all, we showed that AL-9 is an inhibitor of purified type III PI4 kinases. Furthermore, we clearly demonstrated that AL-9 inhibits PI4KIIIα both in naïve Huh7.5 cells (Figure 5, discussed above) as well in cells harboring actively replicating HCV RNA (Figure 8). In cells where HCV replication occurs, PI4KIIIα interacts physically with HCV NS5A. This interaction, in turn, leads to the stimulation of PI4P synthesis at the HCV replication sites [32]. Treatment of replicon-harboring cells with AL-9 leads to efficient suppression of the PI4P pool at the HCV replication sites and does so independently of inhibition of HCV replication. This indicates that – although the enzymatic activity of PI4KIIIα is modulated by the interaction with the HCV protein NS5A – it remains sensitive to the action of the 4-anilino quinazoline inhibitor.

We also investigated whether the dramatic changes observed in PI4P membrane levels by treatment with AL-9 could be associated with alteration in the subcellular distribution of type III PI4 kinases. To this aim, we analyzed the subcellular distribution of the type III PI4 kinases in Huh7.5 or Luc-A4 cells following incubation with AL-9 (Figure S3). We observed no major effect of AL-9 on the localization of either PI4KIIIα or PI4KIIIβ, in line with the notion that the observed effects are primarily due to the inhibition of the kinase activity rather than to an altered protein subcellular distribution.

In cells that express the HCV polyprotein from a trans-gene, knock-down of PI4KIIIα by RNAi was previously shown to cause a dramatic change in NS5A subcellular distribution, from a pattern consistent with localization in the membranous web replication complexes to abnormally large cytoplasmic clusters [27], [30], [32]. In Figure 9, we show that AL-9 treatment of cells ectopically expressing the HCV nonstructural proteins results in a time-dependent depletion of PI4P and a concomitant change of NS5A localization to the large-clustered structures discussed above, reinforcing the notion that the anti-HCV effect of AL-9 and related compounds are likely to be mediated by the inhibition of PI4KIIIα.

We also found that PI3K p110α is inhibited by AL-9 in vitro at concentration similar to those needed to inhibit type III PI4-kinases. However, no Class I PI3-kinase has been shown to influence HCV replication thus inhibition of HCV replication by AL-9 is not due to inhibition of Class I PI3-kinases. So far, the only PI3-kinase that resulted as positive hit for HCV replication inhibition in siRNA screens is PI3-kinase C2 gamma [28]. Future work will have to address whether AL-9 inhibits PI3KC2G in addition to Type III PI4-kinases.

During the characterization of AL-9 we focused our attention on various aspects of PI4P metabolism in Huh7.5 cells with and without replicating HCV. We observed a typical Golgi localization of PI4P in intracellular membranes of naïve Huh7.5 cells and confirmed a role for PI4KIIIβ in maintaining at least part of this pool. In order to get the complete picture we also investigated the PI4P pool present in the plasma membrane. In yeast, Stt4p, the ortholog to the mammalian PI4KIIIα, is localized at the plasma membrane and it is the major contributor for the synthesis of the plasma membrane-localized PI4P [43]. In mammalian cells, the role of PI4KIIIα for the maintenance of the plasma membrane PI4P pool has been demonstrated in HEK-293 and Cos-7 cells [35]–[37]. Here we demonstrate that liver-derived Huh7.5 cells are endowed with a rich PI4P pool in the plasma membrane and that the enzyme responsible for its maintenance is PI4KIIIα. In HCV-replicating cells, the subcellular PI4P distribution is profoundly altered. As already reported previously, the presence of HCV causes the induction of a membranous web highly enriched for PI4KIIIα-syntesized PI4P. In accordance, several reports demonstrate that NS5A recruits PI4KIIIα to the membranous web by direct protein-protein interaction, thereby stimulating its enzymatic activity [30]–[32]. Concomitantly with the induction of highly PI4P-enriched internal membranes, we observe a marked decrease of PI4P in the plasma membrane. One possible explanation could be that – by hijacking PIKIIIα – HCV might be able to enrich PI4P in the virus-induced membranous web not only by directly activating the enzymatic activity of PI4KIIIα recruited into the HCV RNA replication compartment, but also by preventing transport of the PI4KIIIα-synthesized PI4P from the synthesis site to the plasma membrane. How PI4KIIIα, localized at the ER, synthesizes the PI4P pool present in the plasma membrane it is still an enigma. This topological discrepancy can partially be resolved assuming that PI4KIIIα-dependent PI4P production occurs on ER-PM contact sites, that is, sites of close apposition between ER and PM. In yeast it has been demonstrated that a complex interplay between different proteins regulate the PI4P metabolism at the plasma membrane [47]. Among these proteins are Osh, the yeast ortholog of the human OSBP and the ER membrane VAP proteins Scs2 and Scs22, the yeast orthologs of human VAP proteins. Interestingly, h-VAP-33 and OSBP have been shown to be important for HCV replication [48]–[50]. It may be possible that recruitment of PI4KIIIα to the HCV membranous web through NS5A prevents interaction of PI4KIIIα with its cellular protein partners required to direct PI4P to the plasma membrane. Upon withdrawal of HCV from the cells (Figure 7) PI4KIIIα is again free for interaction with the adequate partners. A possible role of PI4KIIIα in PI4P trafficking between the plasma- and intracellular membranes is suggested by our finding that RNAi silencing of this PI4 kinase results in decreased concentration of PI4P in the plasma membrane with a concomitant increase in the level of PI4P in the endomembranes (Figure 3). Such a function of PI4KIIIα would have to be independent of the kinase activity, since pharmacological inhibition (with PIK93 or AL-9) does not recapitulate this phenomenon observed by knocking down the protein expression.

In summary, the presence of HCV may change PI4P metabolism not only by activating the catalytic activity of PI4KIIIα by NS5A but also by modulating the PI4P distribution between different membrane compartments. The net result is an enrichment of the PI4P pool in the HCV-induced membranous web with a concomitant depletion of the plasma membrane PI4P pool.

Concluding, in this paper we demonstrate that a class of HCV inhibitors originally proposed to target NS5A does in fact target the host factor PI4KIIIα. Compounds targeting host factors may have the general advantage of imposing a higher genetic barrier to the development of resistance. AL-9, a member of this class of compounds, inhibits PI4KIIIα and to our knowledge, it is the first compound with a clear preference for PI4KIIIα over PI4KIIIβ. For this reason, AL-9 offers a good candidate as lead compound for the development of more potent and specific pharmacological inhibitors of PI4KIIIα to be used both as important research tools as well as leads for initial drug discovery.

## Materials and Methods

### Reagents and plasmids

The HCV RNA polymerase inhibitor HCV-796 and the PI kinase inhibitor PIK93 were a gift from Arrow Pharmaceuticals. The HCV protease inhibitor MK-5172 was purchased from Selleck Chemicals. Nucleic acids were manipulated according to standard protocols. Plasmid FBac-His-CD-PI4KA was constructed as follows: the catalytic domain of PI4KIIIα was amplified by PCR using the oligonucleotides 5′-CACTGCGGATCCATAATGGGGATGATGCAGTGTGTGATTG-3′ (sense), 5′-CCTGCGAATTCTCAGTAGGGGATGTCATTC-3′ (antisense) and the plasmid pEF1A-PIK4CA untagged (a kind gift from G. Randall, Department of Microbiology, University of Chicago) as template. The resulting PCR fragment was subcloned into the vector pCR-Blunt II-Topo (Invitrogen) and finally cloned into the BamH1–XhoI cloning sites of the plasmid vector pFastBac THT-B. The resulting protein expressed from this plasmid contains an N-terminal hexa-histidine tag and starts at PI4KIIIα amino acid G873 (reference sequence NM_058004). pTM-NS3-5B expression vector expressing the HCV genotype 2a nonstructural proteins under the control of the T7 promoter was a generous gift from V. Lohmann (Department of Molecular Virology, University of Heidelberg) [42]. Synthesis of compound AL-9 is described in **Supporting Information**.

### Cells lines and culture conditions

The human hepatoma-derived cell line Huh7.5 [51] were grown in Dulbecco's modified Eagle's medium (DMEM) supplemented with 10% fetal bovine serum, 100 U/ml penicillin, 100 µg/ml Streptomycin and 2 mM L-glutamine; G418 (0.8 mg/ml) was added to cell lines containing the HCV replicon. Stable cell lines expressing HCV genotype 1b or 2a subgenomic replicons were generated by electroporation of in vitro-transcribed RNA into Huh7.5 cells [52] and following selection with G418 (0.8 mg/ml) for three weeks. Con1-SR: Huh7.5 cells replicating the Con1 subgenomic replicon with the adaptive mutations E1202G in NS3 and S2204R in NS5A. JFH-A4: Huh7.5 cells replicating the JFH-1 subgenomic replicon together with the luciferase reporter gene constructed as described previously [53]. JFH-A4 cells were cured from the HCV replicon by two weeks of treatment with the protease inhibitor MK-5172 (0.2 µM) or the HCV RNA polymerase inhibitor HCV-796 (2 µM), respectively. Huh7-Lunet/T7 cells were a kind gift from V. Lohmann (Department of Molecular Virology, University of Heidelberg, Germany).

### Replication and infection assays

For replication assays, JFH-A4 or Con1-SR cells were plated at the density of 3×10^4^ or 6×10^4^ cells/well, respectively, in 24-well dishes the day before the experiment. Cells were treated with AL-9 resulting in a final concentration of 1% DMSO in the cell medium. After three days of treatment, RNA was extracted using the RNeasy Mini Kit (Qiagen) and HCV RNA was quantified by real time PCR using the following oligonucleotide and probe set designed for the HCV IRES as described previously [52]: sense (5′-GCGAAAGGCCTTGTGGTACT-3′), antisense (5′-CACGGTCTACGAGACCTCCC-3′), and probe (5′-CCTGATAGGGTGCTTGCGAGTGCC-3′, 5′ 6-carboxyfluorescein [FAM]/3′ 6-carboxytetramethylrhodamine [TAMRA]). GAPDH mRNA was used as internal control for data normalization.

Production of infectious virus was performed as follows: J6/JFH-1 chimeric RNA (1-846(J6CF)/847-3034(JFH1) was electroporated into Huh7.5 cells using the protocol described previously [52]. Briefly, 2×10^6^ cells were electroporated with 10 µg of RNA in a final volume of 200 µl and 4×10^6^ cells were plated in a T-75 flask. Three days post electroporation, medium was harvested and stored at −20°C in small aliquots. Calculation of EC_50_ of AL-9 using the infectious HCV virus was performed as follows: Huh7.5 cells were plated at 4×10^4^ cells/well in 24-well plates the day before infection. Infection was started by addition of 10 µl of cell medium containing infectious virus (see above) at an MOI of 50 in a final volume of 400 µl. After 6 hours of incubation, medium was removed and replaced with 400 µl of fresh medium containing serial dilutions of AL-9. RNA was collected after 72 hours of incubation and quantified by real time PCR. Cell cytotoxicity (CC_50_) of AL-9 was calculated using the cell viability assay CellTiter-Blue (Promega). Huh7.5, JFH-A4, or Con1-SR cells (5×10^3^ cells/well in 96-well dishes) were plated the day before treatment. AL-9 was added and cell viability was measured after four days of treatment.

### Expression and purification of the catalytic domain of PI4KIIIα

Recombinant baculovirus was generated with the plasmid FBac-His-CD-PI4KA using the Bac-to-Bac system following the instructions of the manufacturer (Invitrogen). For protein expression, Sf9 cells were infected with recombinant baculovirus at a density of 2×10^6^ Sf9 cells/ml for 3 days at 20°C. To prepare cell extract (1.5×10^8^ cells), cells were incubated in hypotonic buffer (10 mM HEPES (pH 7.5), 10 mM NaCl, 1 mM Tris(2-carboxyethyl)phosphine (TCEP) and EDTA-free protease inhibitor cocktail (Complete, Roche) for 30 min in ice and mechanically broken by 20 strokes of a Dounce homogenizer. After homogenizing, cells were incubated in lysis buffer (50 mM HEPES (pH 7.5), 500 mM NaCl, 10% glycerol, 1% Triton-X100, 1 mM TCEP and EDTA-free protease inhibitor cocktail (Complete, Roche) for further 30 min in ice and cell extract was cleared by centrifugation for 45 min at 20.000 g. The cleared supernatant was incubated in batch with Ni-Sepharose High Performance (GE Healthcare) for 2 hours at 4°C with continuous shaking. The resin was first washed with 10 resin-volumes of wash buffer (50 mM HEPES (pH 7.5), 10% glycerol, 0.4% Triton X-100, 150 mM NaCl and 20 mM imidazol) followed by elution with wash buffer containing 250 mM imidazole. Active fraction (0.5 ml) were dialyzed against 50 mM HEPES (pH 7.5), 150 mM NaCl, 1 mM DTT, 0.4% Triton X-100 and 10% glycerol and stored at −80°C in small aliquots.

### In vitro kinase assay

PI4K kinase activity was assayed with the ADP-Glo Kinase Assay (Promega), according to the manufacturer's instructions. Briefly, 0.5 µl of PI4KIIIα-CD or 0.05 µl PI4KIIIβ (32 ng, Invitrogen) were preincubated with DMSO or AL-9 in reaction buffer (20 mM Tris (pH 7.5), 5 mM MgCl_2_, 2 mM DTT, 0.5 mM EGTA, 0.4% Triton X-100) for 10 min at room temperature in a final volume of 8 µl. The reaction was started by addition of 2 µl of ATP and PI∶PS Lipid Kinase Substrate (Invitrogen) to give a final concentration of 100 µM and 150 µM, respectively. After 1 hour of incubation at room temperature the reaction was stopped and further processed as described by the manufacturer. In parallel the reaction was performed without PI∶PS substrate in order to detect contaminating ATPase activity present in the protein fractions. This activity was subtracted from the measured kinase activity. Kinase activity of PI3Kα (p110α/p85α) and PI3Kβ (p110β/p85α) was assayed as above using 5 ng or 20 ng, respectively (Millipore). Reaction buffer was changed to 50 mM HEPES pH 7.5, 10 mM MgCl_2_ and 1 mM DTT.

### Indirect immunofluorescence

Cells were plated one day before the experiment in 24-well plates (5×10^4^ cells/well for Huh7.5 and JFH-A4 cells, 7×10^4^ cells/well for Con1-SR and 1×10^5^ cells/well for cured JFH-A4 cells). Cells were either untreated or treated with compounds for the time as indicated in the figure legend. PI4P staining of the plasma membrane or internal membranes was performed exactly as described previously [37]. Primary antibodies used were: anti-PI4P IgM (Cat.No. Z-P004, 1∶300, Echelon), anti-Giantin antibody (Cat. No. PRB-114C-200 1∶1000, Covance), affinity-purified rabbit anti-NS5A antibody (1∶2000) [54], anti-PI4KIIIα kinase (Cat. No. 4902, 1∶50, Cell Signaling), anti-PI4KIIIβ kinase (Cat. No, 611817, 1∶500, BD Transduction). Secondary antibodies used were goat anti-mouse IgM Alexa Fluor 488 (Cat. No. A-21042, 1∶600, Invitrogen) and goat anti-rabbit Alexa Fluor 568 (Cat.No. A-11011, 1∶600, Invitrogen). For type III PI4K kinases or NS5A staining, all incubations were performed at room temperature. Cells were washed once with PBS and fixed with 300 µl of 4% PFA for 15 min. Cells were washed three times with PBS and permeabilized with 500 µl of 0.1% Triton X-100 (or 0.5% for PI4KIIIα kinase staining) in PBS for 10 min. Unspecific binding was blocked by incubation with 3% BSA in PBS (for PI4KIIIα staining no blocking was performed). After incubation with the primary antibody in blocking buffer, cell were washed with PBS and subsequently incubated with goat secondary antibodies conjugated to Alexa-Fluor 568, or Alexa-Fluor 488 at a dilution of 1∶600. Nuclei were stained with Hoechst dye 33342 (Sigma; 1∶4000). Slides were then mounted with 5 µl ProLong Gold Antifade (Invitrogen) and analyzed by using an inverted Leica TCS SP5 scanning laser confocal microscope. Digital images were taken using LAS AF software (Leica) and processed using Volocity software (Perkin Elmer). Quantification of fluorescence intensity was determined from multiple images using Volocity. Relative changes in fluorescence intensity mean values where obtained from four randomly picked fields for each condition (150∼300 cells). For plasma membrane staining, total PI4P fluorescence intensity obtained in each condition was normalized to the number of cells present in each field. For the quantification of relative PI4P levels in internal membranes, PI4P fluorescence intensity was normalized using the fluorescence intensity of the Golgi marker giantin. Quantitative immunofluorescence data are presented as means ± the standard error of the mean (SEM). For the calculation of statistical significance, a two-tailed, unpaired t-test was performed.

### siRNA silencing

3×10^4^ Huh7.5 cells/well were seeded in 24-well plates on microscope cover glasses and transfected with 50 nM of siRNAs in serum-free Opti-MEM (Invitrogen) using Lipofectamine RNAiMAX (Invitrogen), according to the manufacturer's protocol. For western blot analysis, the transfection reaction was proportionally scaled up to 6-well plates. In order to maximize the silencing efficiency, 24 hours after the first transfection, the cells were subjected to a second round of siRNA transfection. siRNA sequences were the following (5′→3′ sense strand): mock siRNA, 5′-GUAUGACCGACUACGCGUA[dT][dT]-3′ (custom, Sigma-Aldrich); PI4KIIIα siRNA, 5′-CCGCCAUGUUCUCAGAUAA[dT][dT]-3′ (custom, Sigma-Aldrich); and PI4KIIIβ siRNA, 5′-GCACUGUGCCCAACUAUGA[dT][dT]-3′ (Silencer Validated siRNA s10543; Ambion). Three days after the initial transfection, cells were stained for PI4P as described previously [37], or subjected to western blot analysis. For immunoblot analysis of protein expression, cells were harvested with TEN buffer (10 mM Tris/HCl pH 8.0, 1 mM EDTA, 100 mM NaCl), washed once with PBS and lysed with 2X protein sample buffer (125 mM Tris-HCl pH 6.8, 10 mM EDTA, 0,003 gr bromophenol blue, 20% glycerol, 4% SDS and 10% β-mercaptoethanol; 200 µl). The samples were then sonicated, heated at 95°C and loaded onto 7.5% polyacrylamide-SDS page (Criterion, Biorad). After electrophoresis proteins were transferred to a nitrocellulose membrane and unspecific binding was blocked by PBS supplemented with 0.5% Tween (PBS-T) and 5% milk. Membranes were then incubated overnight at 4°C with primary antibodies (anti-PI4KIIIα, cat no. 4902, 1∶250 Cell Signaling, anti-PI4KIIIβ, cat. No. 611817, 1∶3000 BD Transduction Laboratories, mouse anti-β-actin, cat. No. A1978, 1∶5000, Sigma). HRP-conjugated secondary antibodies (donkey anti-rabbit, Cat. No. 9341 and sheep anti-mouse, Cat. No. 9311, GE Healthcare) were incubated for 1 hour at room temperature and detection was performed using SuperSignal-Femto chemiluminescent substrate (Pierce-Thermo Scientific).

### T7-driven HCV polyprotein expression

1.5×10^6^ Huh7-Lunet/T7 cells/100 mm dish were transfected with 20 µg pTM-NS3-5B using the transfection reagent Lipofectamine 2000 (Invitrogen). Six hours after transfection, cells were seeded in 24-well plates on microscope cover glasses for indirect immunofluorescence. After 5 hours, cells were treated either with DMSO or with 8 µM AL-9 for 2, 8 or 16 hours and co-staining of NS5A and PI4P was performed using the Golgi staining protocol, as described previously [37].

## Supporting Information

Figure S1 **Synthetic pathway for compound AL-9.** Reagents and conditions: (a) n-BuLi, dry THF, −78°C 1 h, 20°C 3 h, Bu_3_SnCl, −78°C 2 h, RT overnight; (b) Formamide, 155°C, 16 h; (c) SOCl_2_, dry DMF, reflux, 5 h, 4-morpholinoaniline, dry CH_3_CN, reflux, 16 h; (d) compound 1, bis(triphenylphosphine) palladium dichloride, dry THF, reflux; (e) HCl 2M, THF/H_2_O 1 ∶ 1, RT; (f) NaBH(OAc)_3_, CH_2_Cl_2_/AcOH (15∶1), RT.
(TIF)

Figure S2 **HCV replicons harboring putative 4-anilino quinazoline resistance mutations retain sensitivity to inhibition by AL-9.** Huh7.5 cells where transiently transfected with genotype 1b subgenomic replicons carrying mutation triplets reported to be associated to resistance to 4-anilino quinazolines (ET-FAG, ET-PPA or ET-DLD) or with the parental replicon (ET). The ET replicon is a derivative of the Con-1 replicon that contains adaptive mutations at positions E1202G, T1280I, and K1846T [1], *i.e*, the same genetic background used in the original resistance study [2]. The putative resistance mutations triplets engineered in this replicon were as follows: ET-FAG (L199F, V362A, S390G in NS5A); ET-PPA (T200P, S370P in NS5A and S76A in NS5B); ET-DLD (E212D, P299L, V388D in NS5A). Transfected cells were treated with AL-9 for three days. Inhibitory dose-response curve of AL-9 are shown. Transient HCV replication was measured by Luciferase activity and is expressed as % of the DMSO control. The data are averages from of three experimental replicates. EC_50_ values +/−1 SD are shown in the figure inset. Replicon ET-FAG did not replicate at appreciable levels.
(TIF)

Figure S3 **Effect of AL-9 on subcellular distribution of type III PI kinases.** Cellular localization of PI4KIIIα (green), PI4KIIIβ (green) or NS5A (red) was analyzed by immunofluorescence in Huh7.5 or JFH-4A cells incubated for 4 hrs with 8 µM AL-9 or DMSO (control). Zoomed sections are indicated by a white square. No major effect of AL-9 on the localization of either PI4KIIIα or PI4KIIIβ was observed. Under our experimental conditions, we observe very limited colocalization of PI4KIIIα with NS5A (yellow) independent of the treatment with AL-9.
(TIF)

Protocol S1 **Chemical synthesis of compound AL-9.**
(DOC)

Protocol S2 **Construction and assays of HCV replicons harboring putative resistance mutations.**
(DOC)
