# Supplementary figures and images for: Metabolism of Phosphatidylinositol 4-Kinase IIIα-Dependent PI4P Is Subverted by HCV and Is Targeted by a 4-Anilino Quinazoline with Antiviral Activity
Source: PLoS Pathog. 2012 Mar 8;8(3):e1002576. doi: 10.1371/journal.ppat.1002576 (PMC3297592; doi:10.1371/journal.ppat.1002576)

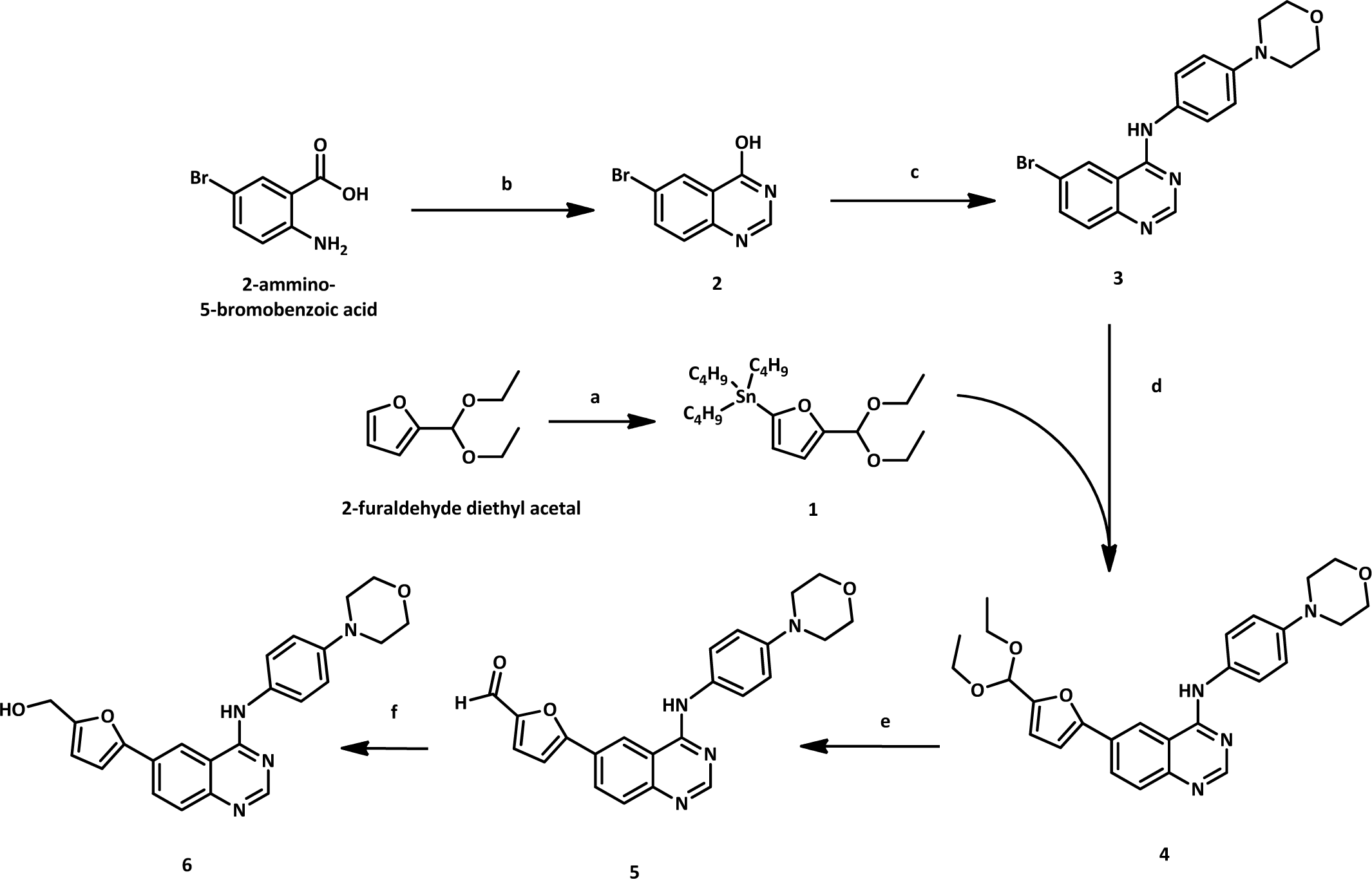

Supplement: Figure S1 — Synthetic pathway for compound AL-9. Reagents and conditions: (a) n-BuLi, dry THF, −78°C 1 h, 20°C 3 h, Bu3SnCl, −78°C 2 h, RT overnight; (b) Formamide, 155°C, 16 h; (c) SOCl2, dry DMF, reflux, 5 h, 4-morpholinoaniline, dry CH3CN, reflux, 16 h; (d) compound 1, bis(triphenylphosphine) palladium dichloride, dry THF, reflux; (e) HCl 2M, THF/H2O 1 ∶ 1, RT; (f) NaBH(OAc)3, CH2Cl2/AcOH (15∶1), RT. (TIF) [file ppat.1002576.s001.tif]

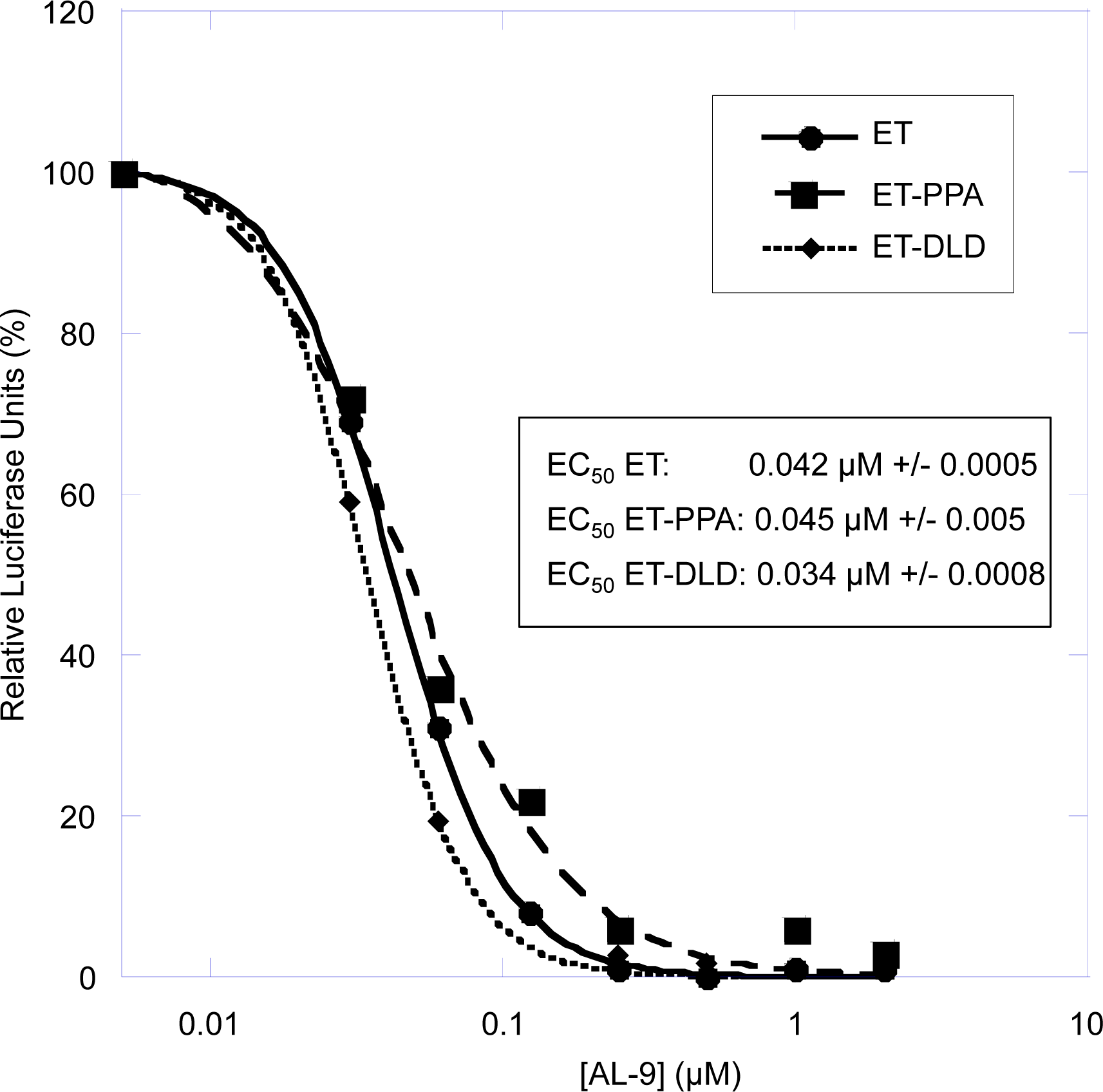

Supplement: Figure S2 — HCV replicons harboring putative 4-anilino quinazoline resistance mutations retain sensitivity to inhibition by AL-9. Huh7.5 cells where transiently transfected with genotype 1b subgenomic replicons carrying mutation triplets reported to be associated to resistance to 4-anilino quinazolines (ET-FAG, ET-PPA or ET-DLD) or with the parental replicon (ET). The ET replicon is a derivative of the Con-1 replicon that contains adaptive mutations at positions E1202G, T1280I, and K1846T [1], i.e, the same genetic background used in the original resistance study [2]. The putative resistance mutations triplets engineered in this replicon were as follows: ET-FAG (L199F, V362A, S390G in NS5A); ET-PPA (T200P, S370P in NS5A and S76A in NS5B); ET-DLD (E212D, P299L, V388D in NS5A). Transfected cells were treated with AL-9 for three days. Inhibitory dose-response curve of AL-9 are shown. Transient HCV replication was measured by Luciferase activity and is expressed as % of the DMSO control. The data are averages from of three experimental replicates. EC50 values +/−1 SD are shown in the figure inset. Replicon ET-FAG did not replicate at appreciable levels. (TIF) [file ppat.1002576.s002.tif]

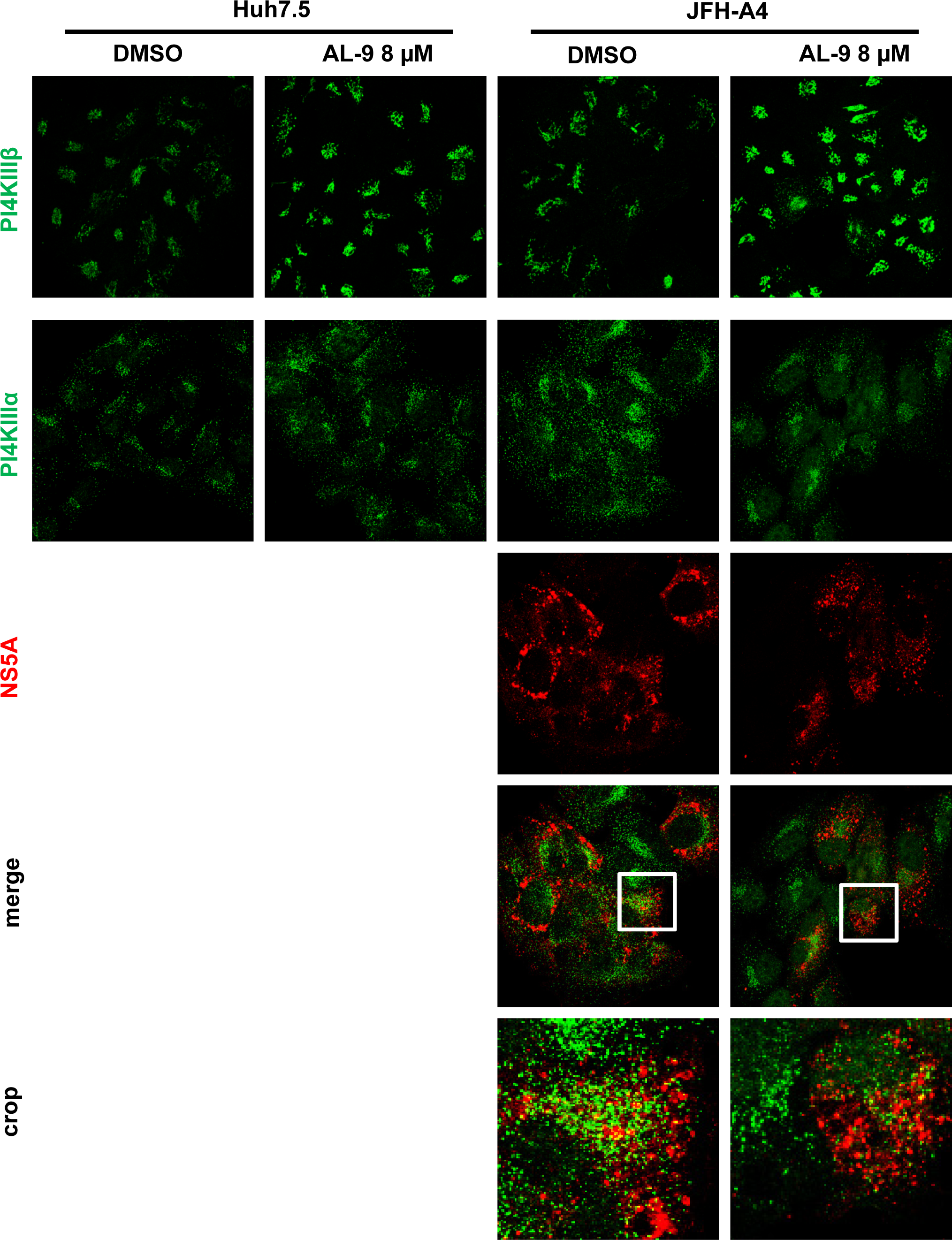

Supplement: Figure S3 — Effect of AL-9 on subcellular distribution of type III PI kinases. Cellular localization of PI4KIIIα (green), PI4KIIIβ (green) or NS5A (red) was analyzed by immunofluorescence in Huh7.5 or JFH-4A cells incubated for 4 hrs with 8 µM AL-9 or DMSO (control). Zoomed sections are indicated by a white square. No major effect of AL-9 on the localization of either PI4KIIIα or PI4KIIIβ was observed. Under our experimental conditions, we observe very limited colocalization of PI4KIIIα with NS5A (yellow) independent of the treatment with AL-9. (TIF) [file ppat.1002576.s003.tif]
