# Supplementary material for: Metabolism of Phosphatidylinositol 4-Kinase IIIα-Dependent PI4P Is Subverted by HCV and Is Targeted by a 4-Anilino Quinazoline with Antiviral Activity
Source: PLoS Pathog. 2012 Mar 8;8(3):e1002576. doi: 10.1371/journal.ppat.1002576 (PMC3297592; doi:10.1371/journal.ppat.1002576)
Supplement: Protocol S1 — Chemical synthesis of compound AL-9. (DOC) [file ppat.1002576.s004.doc]

**Protocol S1**

**Chemical Synthesis of AL-9**

All the reagents were commercially available and used without further purification unless indicated otherwise. All solvents were anhydrous grade unless indicated otherwise. When dry conditions were required, the reactions were carried out in oven-dried glassware under a slight pressure of argon. Reaction were magnetically stirred and monitored by thin-layer chromatography (TLC) on silica gel. TLC was performed on Silica Gel 60 F254 plates (Merck) with UV detection, or using a developing solution of 0.5% orcinol in EtOH/H_2_SO_4_ (3%), followed by heating at 180°C. Flash column chromatography was performed on silica gel 230–400 mesh (Merck). The petroleum ether used as eluent in chromatography has boiling range of 40–60°C. ^1^H and ^13^C-NMR spectra were recorded on a Varian 400 MHz MERCURY instrument at 300 K. Chemical shifts are reported in ppm downfield from TMS as internal standard. Mass spectra were recorded on ESI-MS triple quadrupole (model API2000 QTrap™, Applied Biosystems).

*tributyl-(5-(diethoxymethyl)furan-2-yl)stannane (1)*

To a solution of 2-furaldehyde diethyl acetal (500μL, 2.9 mmol) in diethyl ether (3 mL) cooled to -78°C, *n*-butyllitium (3.9 mmol) was added under Ar atmosphere and the solution was stirred at -78°C for 1h and then for 3 h at 20°C. The solution was cooled again to -78°C and tributyltin chloride (1 mL, 3.9 mmol) was added dropwise; stirring for 2 h at -78°C and 12 h at room temperature.

The reaction was quenched by adding aqueous NH_4_Cl and product extracted with diethyl ether. The organic layer was dried over Na_2_SO_4_ and concentrated *in vacuo*. The crude product was used without any further purification (brown oil, R_f_ = 0.74, AcOEt/hexane 0.2:9.8).

*6-bromoquinazolin-4-ol (2)*

To a solution of 2-ammino-5-bromobenzoic acid (5 g, 23.1 mmol), formamide (15 mL, 377.3 mmol) was added and the mixture was refluxed under Ar atmosphere for 16 h. Then 6 mL of water were carefully added (warning! boiling solution) and product immediately precipitated as a white solid. The mixture was then cooled at RT and 12 mL of water were added slowly. The mixture was stirred 30 min and the resulting precipitate was filtered and re-crystallized from cold ethanol obtaining product (white solid, yield: 53%, R_f_ = 0.51, AcOEt/ petroleum ether 9.4:0.6)

^1^H-NMR (400 MHz; CDCl_3_/CD_3_OD 8:1) δ = 8.34 (d, 1H, J = 2 Hz), 7.97 (s, 1H), 7.83 (dd, 1H, J = 8.7 Hz, J = 2.3 Hz), 7.55 (d, 1H, J = 8.7 Hz).

*6-bromo-N-(4-morpholinophenyl)quinazolin-4-amine (3)*

To a solution of 6-bromoquinazolin-4-ol (2.84 g, 12.6 mmol) in DMF (0.84 mL) thionyl chloride (28 mL) was added and the mixture was refluxed for 5 h. The solvent was evaporated *in vacuo* by adding some toluene (3 x 28 mL) to completely remove thionyl chloride. The residue was dissolved in CH_3_CN (38 mL), 4-morphoaniline (2.48 mg, 13.9 mmol) was added and the mixture was refluxed for 72 h. Crude product precipitated upon cooling the solution and the solid precipitate was recrystallized from diethyl ether obtaining pure product (orange solid, yield: 79%, R_f_ = 0.37, AcOEt).

^1^H-NMR (400 Mhz, DMSO-d_6_) δ = 11.30 (bs, 1H, NH), 9.04 (bs, 1H), 8.87 (s, 1H), 8.19 (bd, 1H, J = 8.8 Hz), 7.79 (d, 1H, J = 8.8 Hz), 7.57-7.04 (AA’XX’ system, 4H, J = 9.0 Hz), 3.74 (m, 4H), 3.14 (m, 4H).

*6-(5-(diethoxymethyl)furan-2-yl)-N(4-morpholinophenyl)quinazolin-4-amine (4)*

To a solution of 6-bromo-N-(4-morpholinophenyl)quinazolin-4-amine (**3**) (300 mg, 0.78 mmol ) in dry THF (7 mL), tributyl-(5-(diethoxymethyl)furan-2-yl)stannane (**1**) (1.4 g, 3.1 mmol) and bis(triphentlphosphine) palladium dichloride (44 mg, 0.06 mmol) were added. The reaction mixture was heated to reflux for 25 h under Ar atmosphere. The solvent was evaporated *in vacuo*, the crude product was dissolved in AcOEt and washed with water and brine. The organic layer was dried over Na_2_SO_4_ and concentrated *in vacuo*. The crude product obtained was purified with flash chromatography on silica gel (AcOEt/exane 8:2) obtaining pure product (yellow solid, yield 74%, R_f_ = 0.24, AcOEt /petroleum ether 9.4:0.6).

^1^H-NMR (400 MHz, CDCl_3_) δ= 8.65 (s, 1H), 8.20 (d, 1H, J = 1.5 Hz), 7.97 (dd, 1H, J = 8.8 Hz, J=1.5 Hz), 7.84 (d, 1H, J = 8.8 Hz), 7.56-6.93 (AA’XX’ system, 4H, J = 8.5 Hz), 6.70 (d, 1H, J = 2.7 Hz), 6.53 (d, 1H, J = 2.7 Hz), 5.57 (s, 1H), 3.87 (m, 4H), 3.60 (m,4H), 3.13 (m, 4H), 1.25 (m, 6H).

^13^C-NMR (400 MHz, CDCl_3_) δ = 158.02, 155.05, 152.70, 151.97, 149.30, 148.73, 130.24, 129.03, 128.89, 128.48, 124.21, 116.19, 115.31, 114.91, 110.71, 106.86, 96.27, 66.88, 61.54, 49.51, 15.15.

ESI-MS: calculated 474.34, found: 475.21 [M+H^+^]; 497.21 [M+Na^+^].

*5-(4-((4-morpholinophenyl)amino)quinazolin-6-yl)furan-2-carbaldehyde (5)*

To a solution of 6-(5-(diethoxymethyl)furan-2-yl)-*N*-(4-morpholinophenyl)quinazolin-4-amine (**4**) (87 mg, 0.18 mmol) in THF (8 mL), HCl 2M (1.6 mL) was added and the mixture was reacted for 2 h at room temperature. The reaction mixture was basified to pH 8 with NaOH 2M. Organic solvent was evaporated *in vacuo* and the aqueous residue was extracted with AcOEt. The organic layer was dried over Na_2_SO_4_ and concentrated *in vacu*o. Product (orange solid, yield 98%, R_f_ = 0.61, AcOEt /MeOH 9:1) was used without any further purification.

^1^H-NMR (400 MHz, CDCl3) δ = 9.60 (s, 1H), 8.65 (1H, s), 8.41 (1H, d, J =1.3 Hz), 8.01 (dd, 1H, J= 8.8 Hz, 1.5 Hz ), 7.85-7.57 (AA’XX’ system, 4H, J = 8.8 Hz), 7.32 (d, 1H, J = 3.7 Hz), 6.92 (m, 3H), 3.82 (t, 4H, J = 4.8 Hz), 3.11 (t, 4H, J = 4.8 Hz).

^13^C-NMR (400 MHz, CDCl_3_) δ = 177.21, 158.30, 156.05, 152.12, 150.55, 148.81, 130.03, 129.35, 129.31, 126.35, 124.28, 117.92, 116.03, 115.47, 108.67, 66.84, 49.39.

ESI-MS: calculated 400.15, found: 401.13 [M+H^+^].

*(5-(4-((morpholinophenyl)amino)quinazolin-6-yl)furan-2-yl)methanol (6)*

To a solution of 5-(4-((4-morpholinophenyl)amino)quinazolin-6-yl)furan-2-carbaldehyde (**5**) (140 mg, 0.35 mmol) in dry CH_2_Cl_2_ (5mL) and AcOH glacial (0.3 mL), sodium triacetoxyborohydride (148 mg, 0.70 mmol) was added. The solution was reacted for 3 h at room temperature. Fresh CH_2_Cl_2_ was added and washed with water and brine. Aqueous layer was washed again with fresh AcOEt. The combined organic layers were dried over Na_2_SO_4_ and concentrated *in vacuo*. The crude product obtained was purified with flash chromatography on silica gel (AcOEt) obtaining pure product (yellow solid, yield 35%, R_f_ = 0.53 AcOEt/MeOH 9:1).

^1^H-NMR (400 MHz, DMSO-d_6_) δ = 9.84 (s, 1H), 8.74 (d, 1H, J = 1.4 Hz), 8.44 (s, 1H), 8.11 (dd, 1H, J = 8.8 Hz, J = 1.4 Hz), 7.74-7.61 (AA’XX’ system, 4H, J = 8.8 Hz), 7.03 (d, 1H, J = 3.2 Hz), 6.98 (d, 2H, J = 8.8 Hz), 6.48 (d, 1H, J = 3.2 Hz), 5.32 (t, 1H, J = 5.7 Hz), 4.50 (d, 2H, J = 5.7 Hz), 3.74 (t, 4H, J = 4.7 Hz), 3.09 (t, 4H, J = 4.7 Hz).

^13^C-NMR (100 MHz, DMSO-d_6_) δ= 157.80, 156.04, 154.53, 151.79, 148.88, 147.90, 130.79, 128.36, 128.09, 124.14, 116.59, 115.43, 115.07, 109.59, 107.65, 66.13, 55.84, 48.81.

ESI-MS: calculated, 402.17, found: 403.19 [M+H^+^].
