# Supplementary material for: Metabolism of Phosphatidylinositol 4-Kinase IIIα-Dependent PI4P Is Subverted by HCV and Is Targeted by a 4-Anilino Quinazoline with Antiviral Activity
Source: PLoS Pathog. 2012 Mar 8;8(3):e1002576. doi: 10.1371/journal.ppat.1002576 (PMC3297592; doi:10.1371/journal.ppat.1002576)
Supplement: Protocol S2 — Construction and assays of HCV replicons harboring putative resistance mutations. (DOC) [file ppat.1002576.s005.doc]

**Protocol S2**

**Construction and assays of HCV replicons harboring putative resistance mutations**

The mutants pFKi341-PiLuc-NS3-3’/ET-DLD (E212D, P299L, V388D in NS5A) and pFKi341-PiLuc-NS3-3’/ET-FAG (L199F, V362A, S390G in NS5A) were generated starting from pFKi341-PiLuc-NS3-3’/ET (provided by V. Lohman). Two synthetic fragments (737 or 767 bp, respectively) containing the indicated triple mutations were provided from MWG-Eurofins. Using Crossover PCR-based method SalI-BamHI fragments carrying mutations were obtained and transferred into pFKi341-PiLuc-NS3-3’-ET backbone. The mutant pFKi341-PiLuc-NS3-3’-ET-PPA (T200P, S370P in NS5A and S76A in NS5B) was obtained using Multi Site-Directed Mutagenesis Kit (Agilent Technologies). In particular, the MluI-SpeI fragment of pFKi341-PiLuc-NS3-3’-ET was subcloned into pCR2.1 vector, mutated according to manifacturer’s protocol and transferred back into the pFKi341-PiLuc-NS3-3’-ET backbone. All numbers refer to aminoacid position of HCV proteins (HCV Con-1; EMBL database accession number AJ238799).

The pFKi341-PiLuc-NS3-3’/ET construct and the mutants pFKi341-PiLuc-NS3-3’/ET-DLD, pFKi341-PiLuc-NS3-3’/ET-FAG and pFKi341-PiLuc-NS3-3’/ET-PPA were linearized at the 3’ end of the viral genome by ScaI digestion and purified by Sodium Acetate precipitation. RNA transcripts were generated and purified by MEGAscript T7 kit (Ambion) according to the manufacturer’s protocol. 2x10^6^ Huh7.5 cells were electroporated with 10 μg of each RNA construct in a final volume of 200 μl as described previously [3]. Electroporated cells were plated at the density of 7.5x10^3^ cells/well in 96-well plates. 6 hours after electroporation, cells were treated with serial dilutions of AL-9. After three days of treatment, cell viability was measured by CellTiter-Blue® (Promega) and luciferase activity was measured by Bright-Glo™ Luciferase Assay System (Promega), according to the manufacturer’s protocols.

**References**

1. Krieger N, Lohmann V, Bartenschlager R (2001) Enhancement of hepatitis C virus RNA replication by cell culture-adaptive mutations. J Virol 75: 4614-4624.

2. Najarro P, Powell K, Budworth A, Hallott A, Harris R, et al. (2006) A-831, a novel HCV inhibitor targeting NS5A. 1st International Workshop on Hepatitis C - Resistance & New Compounds. 25-26 October 2006, Boston, U.S.A.

3. Pacini L, Graziani R, Bartholomew L, De Francesco R, Paonessa G (2009) Naturally occurring hepatitis C virus subgenomic deletion mutants replicate efficiently in Huh-7 cells and are trans-packaged in vitro to generate infectious defective particles. J Virol 83: 9079-9093.
